# Structure and Molecular Recognition Mechanism of IMP-13 Metallo-β-Lactamase

**DOI:** 10.1128/AAC.00123-20

**Published:** 2020-05-21

**Authors:** Charlotte A. Softley, Krzysztof M. Zak, Mark J. Bostock, Roberto Fino, Richard Xu Zhou, Marta Kolonko, Ramona Mejdi-Nitiu, Hannelore Meyer, Michael Sattler, Grzegorz M. Popowicz

**Affiliations:** a Biomolecular NMR and Center for Integrated Protein Science Munich at Department Chemie, Technical University of Munich, Garching, Germany; b Institute of Structural Biology, Helmholtz Zentrum München, Neuherberg, Germany; c Department of Biochemistry, Faculty of Chemistry, Wroclaw University of Science and Technology, Wroclaw, Poland; d Institute for Medical Microbiology, Immunology and Hygiene, Technical University of Munich, Munich, Germany

**Keywords:** IMP-13, metallo-β-lactamase, imipenemase, antibiotic resistance, solution NMR, X-ray crystallography, molecular dynamics, metalloenzyme, protein dynamics, β-lactam antibiotic, nuclear magnetic resonance

## Abstract

Multidrug resistance among Gram-negative bacteria is a major global public health threat. Metallo-β-lactamases (MBLs) target the most widely used antibiotic class, the β-lactams, including the most recent generation of carbapenems. Interspecies spread renders these enzymes a serious clinical threat, and there are no clinically available inhibitors. We present the crystal structures of IMP-13, a structurally uncharacterized MBL from the Gram-negative bacterium Pseudomonas aeruginosa found in clinical outbreaks globally, and characterize the binding using solution nuclear magnetic resonance spectroscopy and molecular dynamics simulations. The crystal structures of apo IMP-13 and IMP-13 bound to four clinically relevant carbapenem antibiotics (doripenem, ertapenem, imipenem, and meropenem) are presented. Active-site plasticity and the active-site loop, where a tryptophan residue stabilizes the antibiotic core scaffold, are essential to the substrate-binding mechanism. The conserved carbapenem scaffold plays the most significant role in IMP-13 binding, explaining the broad substrate specificity. The observed plasticity and substrate-locking mechanism provide opportunities for rational drug design of novel metallo-β-lactamase inhibitors, essential in the fight against antibiotic resistance.

## Abstract (precis)

Multidrug resistance among Gram-negative bacteria is a major global public health threat. Metallo-β-lactamases (MBLs) target the most widely used antibiotic class, the β-lactams, including the most recent generation of carbapenems. Interspecies spread renders these enzymes a serious clinical threat, and there are no clinically available inhibitors. We present the crystal structures of IMP-13, a structurally uncharacterized MBL from the Gram-negative bacterium Pseudomonas aeruginosa found in clinical outbreaks globally, and characterize the binding using solution nuclear magnetic resonance spectroscopy and molecular dynamics simulations.

## INTRODUCTION

Multidrug-resistant bacteria pose a major challenge to human health, with mechanisms of resistance to all known classes of antibiotics now being identified. While much pharmaceutical research has focused on drugs to treat Gram-positive bacterial infections, multidrug resistance among Gram-negative pathogens remains a significant clinical challenge (1–3). β-Lactam antibiotics are used for the treatment of both Gram-negative and Gram-positive bacterial infections and are the most commonly prescribed antibiotics (4, 5). β-Lactam antibiotics act as inhibitors of cell wall biosynthesis, causing subsequent bacterial cell death (6). The success of the first β-lactam antibiotic, penicillin, discovered in 1928 by Alexander Fleming (7) and used clinically since 1943 (8), led to multiple developments of the β-lactam scaffold, providing new and more effective antibacterial compounds.

As a result of the widespread use of β-lactam antibiotics, resistance mechanisms against them have emerged (3). Resistance mechanisms can be divided into mutation of penicillin binding proteins (PBPs), which prevent the binding of β-lactams to their target protein; reduction of the antibiotic concentration in the cell due to increased efflux (through expression of efflux pumps) or decreased uptake (through altered expression of outer membrane proteins); and, most commonly and significantly, the production of β-lactamase enzymes (9). β-Lactamases hydrolyze the β-lactam ring, which distinguishes this class of antibiotics and which is key to its binding mechanism, thus preventing interaction of the antibiotic with its target (10). As a response to the emergence of enzyme-mediated resistances as early as the 1940s (11), cephalosporin and carbapenem-type β-lactam antibiotics were discovered, isolated, and developed (12–14). Carbapenems are formed of a core scaffold, consisting of a β-lactam ring fused to a pyrroline ring that is decorated with an exocyclic sulfur that links to the tail region of the molecule (see Fig. SI1 in the supplemental material).

However, β-lactamases that are capable of inactivating the most recent generation of carbapenems, often used as a last resort for the effective treatment of infections caused by multidrug-resistant bacteria, have now evolved (15) and spread rapidly (16). As such, carbapenem resistance is a hallmark of all three of the World Health Organization’s highest-priority pathogens (17).

On a structural basis, four main classes of β-lactamases can be defined: three classes of serine β-lactamases, distinguishable by their amino acid sequence and inhibitor susceptibility (Ambler classes A, C, and D), and one class of metallo-β-lactamases (MBLs; Ambler class B), requiring divalent zinc ions for their β-lactamase activity (18). MBLs have been shown to hydrolyze all bicyclic β-lactams, including the carbapenems (19). The existence of MBL genes on integron structures and plasmids, often coexpressed with other antibiotic resistance genes, renders MBLs a serious clinical challenge, due to the possibility of horizontal gene transfer (20, 21). Inhibitors of serine β-lactamases, e.g., clavulanic acid (22) and avibactam (23), are available and used clinically in combination with antibiotics (24). However, resistance to these treatments is already being seen in the clinic (25), and to date, there are no clinically available inhibitors of the class B MBLs, making MBLs a significant threat.

MBLs can be divided into three subclasses (subclasses B1, B2, and B3), based on sequence and structural similarities and the number of coordinated zinc ions, with the B1 class representing the most significant one clinically (26). Among the major B1 class enzymes are the imipenemases (IMP), Verona integron-encoded MBLs (VIM), and the New Delhi MBLs (NDMs), which can hydrolyze the most recent cephalosporins and carbapenems (20, 27, 28).

IMP-type MBLs were first identified in Japan, and the class now consists of at least 53 members (29, 30) identified in more than 26 species of Gram-negative bacteria from around the world (31). IMP-encoding genes have been shown to occur as resistance cassettes along with other resistance genes, including those for serine β-lactamases (32) and those for aminoglycoside (33) and streptomycin (34) resistance. IMPs can be divided into six subgroups based on phylogeny and sequence similarity. IMP-13, a member of subgroup 2, sharing 92.3% amino acid sequence similarity with IMP-2 and 82.5% with IMP-1 (Fig. SI2) (35), was first identified in the Gram-negative pathogen Pseudomonas aeruginosa from clinical samples in Italy (21) and is a common cause of carbapenem resistance, often involved in large outbreaks (36). IMP-13 has been detected in a number of other countries in Europe as well as South America (21, 36–38). While IMP-13 is most commonly associated with Pseudomonas aeruginosa infections, it has also been identified in other human pathogens, including Salmonella enterica; members of the *Enterobacteriaceae*, including *Klebsiella* and *Enterobacter* spp. (38, 39); as well as nonhuman pathogens in the environment e.g., Pseudomonas monteilii, related to the soil microbe P. putida (40). These studies indicate that IMP-13 is present in a variety of significant human pathogens, as well as in other nonhuman pathogens which can act as environmental reservoirs of antibiotic resistance.

The recombinant IMP-13 protein has been overexpressed, purified, and characterized biochemically (41), but no structural information concerning IMP-13 has yet been reported. Crystal structures of MBLs, such as NDM-1 and IMP-1, show a conserved αβ/βα fold, with an active site at the interface of the two αβ units involving one or two zinc ions (42, 43). Although the overall folds are expected to be very similar, divergence between the various structures makes these challenging targets for drug development. Currently, relatively few crystal structures are available for other members of the IMP class, with no structural information on the antibiotic binding mode. Thus, high-resolution structural information is essential to broaden overall knowledge of MBLs and their antibiotic binding modes and enable the design of novel β-lactamase inhibitors to fight antibiotic resistance. The plasticity of the active site is also seen to play a role in other MBL classes (44, 45), so analyzing a wide range of antibiotic binding modes will help to determine the key factors in this.

Here, we report two distinct apo IMP-13 structures and the structures of IMP-13 complexed with four clinically relevant carbapenem antibiotics bound in their hydrolyzed form (doripenem, ertapenem, imipenem, and meropenem). We also present backbone nuclear magnetic resonance (NMR) assignments and NMR relaxation measurements for IMP-13 in the apo and ertapenem-bound forms and molecular dynamics (MD) simulations for the apo and carbapenem-bound states. The structural information and dynamics presented here reveal important information about the mechanism of antibiotic binding, as well as a significant role for the active-site-covering loop (L1), indicating that the plasticity of the active-site region is important for the broad substrate recognition spectrum of these enzymes. The structural information presented here provides important information to further aid in the development of novel MBL inhibitors, essential to combat this significant bacterial threat.

## RESULTS

### Structure of the apo form of IMP-13.

**FIG 1:**
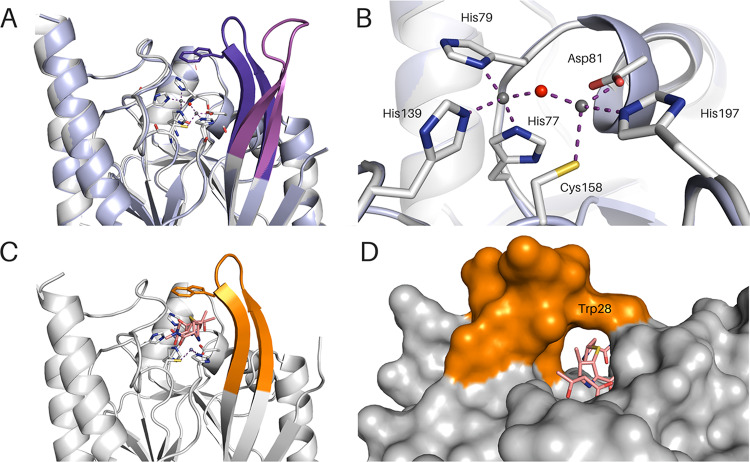
IMP-13 apo and meropenem-bound structures. (A) Overlay of IMP-13 apo structures with open (magenta) and closed (violet) loops. The zinc-coordinating residues of the open state are shown as sticks. (B) Zoomed view of the image in panel A showing coordination of the two Zn(II) ions in the apo structures. (C) IMP-13 meropenem-bound structure. The loop is in orange, and the ligand is in salmon. (D) The closed loop forms a tunnel in the meropenem-bound structure. Zinc ions are shown as gray spheres, and water molecules are shown as red spheres.

Two apo-form crystal structures of IMP-13, showing the L1 active-site loop (Fig. 1; see also Fig. SI3 and 4 in the supplemental material) in the open (apo_open_) and closed (apo_closed_) conformations, were solved to 1.9- and 2.2-Å resolutions, respectively (PDB accession numbers 6R79 and 6R78, respectively). We define the open conformation to be the loop pointing away from the protein toward the solvent and the closed conformation to be the loop positioned over the active site and pointing toward its rim. The distance between C-α atoms of Trp28 for the open and closed conformations is 8.8 Å. The overall protein architecture of the IMP-13 apo structure is consistent with that of the previously published metallo-β-lactamase fold (43), consisting of a global αβ/βα topology with a shallow active-site cleft at the border of the two β-sheets. In the apo structure presenting a closed L1 loop conformation (apo_closed_ conformation, PDB accession number 6R78), the two divalent zinc ions (Zn1 and Zn2) are found at a distance of 3.5 Å apart: one (Zn1) coordinates His77, His79, and His139 residues and a bridging water molecule in a tetrahedral geometry, while the other (Zn2) coordinates Asp81, Cys158, and His197 and the bridging water (Fig. 1B). The bridging water was previously proposed to be in the form of a hydroxide ion for activation of the β-lactam ring for hydrolysis (46, 47) and is seen to be about 3.3 Å from each of the oxygens of the Asp81 side chain, indicating that the hydroxide ion would be oriented by these residues, as seen in IMP-1 (48). In the apo_open_ structure (PDB accession number 6R79), the conformation is determined by interlocking with another loop, while in the closed conformation, these interactions are missing. The B-factor values of the loop residues are, in both cases, approximately 20 Å^3^ higher than the values for the rest of the protein molecule, indicating the high degree of flexibility of this region.

### Structure of carbapenem-bound IMP-13.

**FIG 2:**
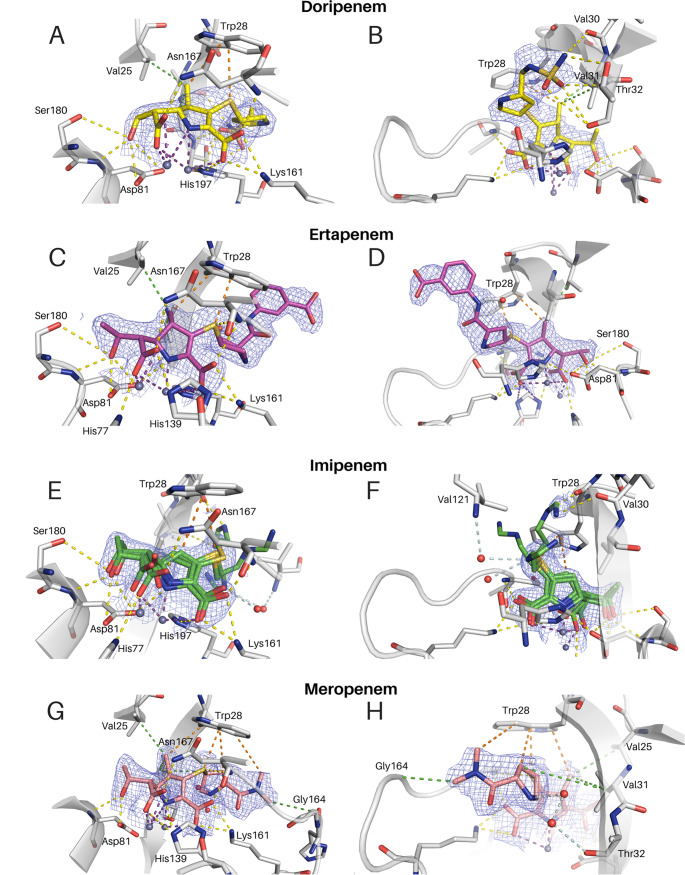
2*F*_o_ − *F*_c_ maps at a contour level of 1σ for the presented complex crystal structures, showing interactions of the antibiotics’ carbapenem scaffold (A, C, E, G) and tails (B, D, F, H) with IMP-13. (E, F) Imipenem (green) shows three distinct tail conformations. All are depicted. (A to D, G, H) Doripenem (yellow) (A, B), ertapenem (magenta) (C, D), and meropenem (salmon) (G, H) are each seen in one conformation in each chain. Interactions are shown as dashed lines. Purple, zinc interactions; yellow, H bonds and charge-charge interactions; orange, aromatic interactions; green, hydrophobic interactions; pale blue, water network. Zinc ions are shown as gray spheres, and water molecules are shown as red spheres.

The crystal structures of IMP-13 bound to hydrolyzed doripenem (2.8 Å, PDB accession number 6S0H), ertapenem (2.2 Å, PDB accession number 6RZS), imipenem (1.9 Å, PDB accession number 6RZR), and meropenem (2.3 Å, PDB accession number 6R73) were solved by molecular replacement, with IMP-13–antibiotic complex crystals being prepared by cocrystallization. Both the tautomers with sp^2^ and sp^3^ carbons at the C-4 position of the carbapenems (46) (all without a hydrogen on N-6) were modeled into the ligand electron density and refined separately. As the resolution of the collected data is moderate and does not allow clear differentiation between the two tautomers and with an understanding that the crystal structure may be a weighted average of the two forms, the tautomer with the lowest B factor, the sp^2^ form, was deemed to be the most representative in all cases (Fig. 2), as C-4 attached to the S is not visibly tetrahedral. From this, with respect to the mechanisms shown by Lisa et al. (49) and Feng et al. (46), we believe that the primary state visible is that of the intermediate EI_2_. According to Lisa et al. (49), this would then become the Δ1 form after addition of the hydrogen via the sulfur-bound carbon atom and so could be a weighted average of these two states. In all the structures, the tail moiety of the carbapenem adopts different positions when bound to chain A or B of the crystal structure. Such an arrangement can be explained by the location of the tail, which experiences crystal packing contacts in one chain and solvent exposure in the other chain, resulting for the latter in high flexibility and rotational freedom, demonstrated by increased B-factor values compared to those for the core atoms of the carbapenem scaffold. Further description focuses on chain B of the doripenem-, ertapenem-, and imipenem-bound structures and chain A of the meropenem-bound structure, where crystal packing is not seen to affect ligand placement.

A comparison of the carbapenem-bound complex structures with apo_closed_ elicits very few distinct differences, with the L1 loop seen to be packed over the antibiotic binding pocket (Fig. 1A, C, and D). The root mean square deviation (RMSD) of the backbone C-α atoms (for 216 out of 217 residues), including the L1 loop, varied from 0.27 to 0.47 Å, showing a high level of structural similarity between the structures, with only a few differences being seen in the flexible loop regions. With the apo_open_ structure, on the other hand, the loop can be seen to point away from the active site, leaving the active site accessible to the substrate (Fig. 1A). The tunnel formation seen in the closed form is completely absent due to the different positioning of both backbone and side chain atoms. In the complex structures, the largest active-site-facing changes seen in the L1 loop occur between residues Val25 and Val31, with these two residues moving toward the carbapenems to form hydrophobic interactions (movements are in the range of 5 Å and 1 Å for Val25 and Val31, respectively). The residues located in the middle of the L1 loop, Val25 and Trp28, show more significant changes, moving approximately 9 to 10 Å in order to cover the substrate during catalysis, thereby closing the tunnel-like structure above the active site of the enzyme and acting as a gatekeeper between the ligand and the solvent. Further conformational differences between the apo and carbapenem-bound structures include the movement of Asn167 closer to the active site in order to facilitate the hydrogen bonding with the substrate (the rest of the L3 loop does not alter its conformation significantly). Strands B7 and B8 are also altered between the open and closed protein conformations, with Tyr123 and Trp124 showing the most pronounced changes.

Compared to the apo structures, the zinc ions in carbapenem-bound IMP-13 are located slightly farther apart, with the distance for the different antibiotics ranging from 3.8 Å (doripenem complex) to 4.2 Å (ertapenem complex), whereas the distance is 3.5 Å for the apo state, presumably to maximize interactions with the ligand. The zinc-protein coordination remains unchanged (Fig. SI5): Zn1 still coordinates the three histidines (His77, His79, and His139), while Zn2 coordinates Asp81, Cys158, and His197, but the bridging water is no longer observed due to the presence of the enzyme’s substrate.

### Conserved binding mode of the carbapenem scaffold to IMP-13.

The binding of the carbapenem scaffold (Fig. 2; Fig. SI1) was similar for the four antibiotics investigated, with contacts to the surrounding residues being created via a network of hydrogen bonds and hydrophobic and electrostatic interactions. In the carbapenem complexes, two loops, L1 and L3 (Fig. 2; Fig. SI3 and 4), interact with the hydrolyzed substrates (Fig. SI6). Both zinc ions show an interaction with N-6 on the pyrroline ring, with Zn2 having a closer interaction (Table S1; Fig. SI5). In addition, there are interactions with carboxylic acid moieties O-9, O-26, and O-27. In all cases, Zn1 coordinates O-9, while Zn2 coordinates O-26 and O-27. Lys161 acts as a counterion to the carboxylate of the carbapenems (O-8, O-9). Hydrogen bonding is observed between the carbapenems’ hydroxyl groups (O-24) and the Asp81 backbone nitrogen. Interactions with the Asp81 side chain and between the side chain of Asn167 and the carbapenems’ hydroxyls (O-26, O-27) are also observed.

**FIG 3:**
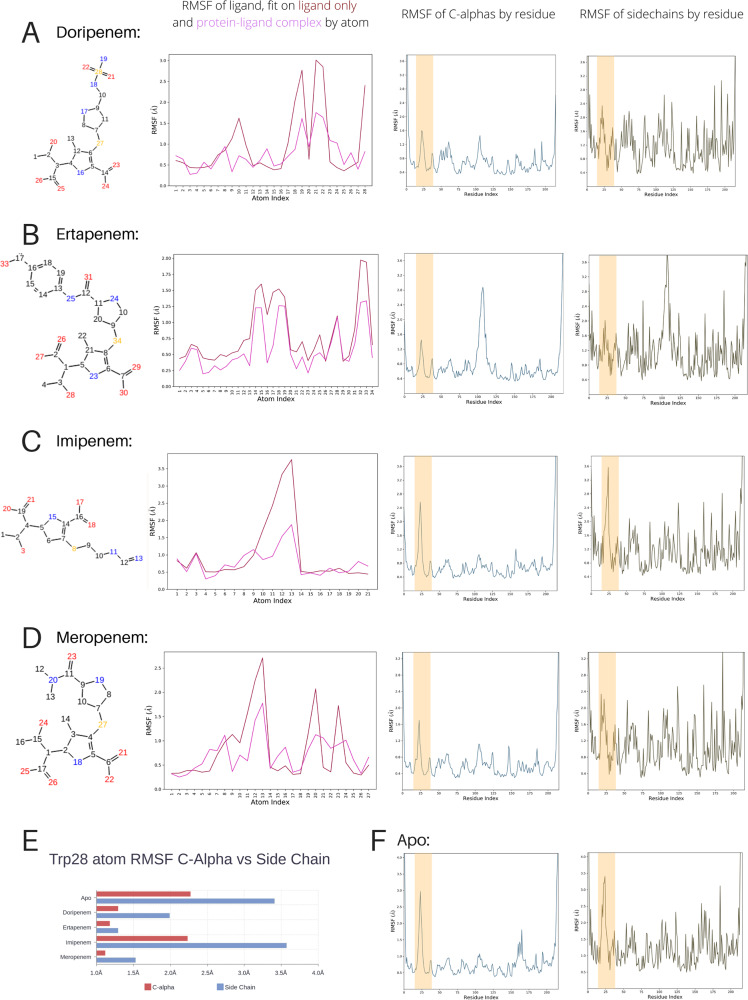
Summary of molecular dynamics simulations for apo and carbapenem-bound IMP-13. The structures of the hydrolyzed antibiotics with the numbering used in the simulations are shown in column 1. Column 2 shows the root mean squared fluctuation (RMSF) of the ligand, fitted on the ligand only and on the protein-ligand complex, column 3 shows the RMSF of the protein C-α atoms, and column 4 shows the RMSF of the protein side chains. The L1 loop is marked by orange bars on all graphs. Results are shown for doripenem (A), ertapenem (B), imipenem (C), and meropenem (D). (F) Values for apo C-α and the side chain RMSF are shown for comparison. (E) C-α and side chain RMSF values for the key residue Trp28 are shown for all structures.

L1, the extended β loop that is conserved in β-lactamases, encompasses the active site, forming a tunnel-like structure of a hydrophobic nature (Fig. 1C and D); the amino acid composition of L1 results in a hydrophobicity index of 0.84 (50), whereas that for the overall protein is −0.32. This largely hydrophobic loop interacts with the β-lactam antibiotics, stabilizing their position during hydrolysis. The tryptophan (Trp28) at the tip of this loop is a key residue that bridges the gap between the loop backbone and the active-site residues, forming a closed tunnel. In IMP-1, the equivalent tryptophan is found to affect *K_m_*: for imipenem, a W64A mutation leads to a 5-fold increase in *K_m_* (51). The sulfur atom present in the linker region (S-10) of all carbapenems creates strong π-sulfur interactions with the aromatic ring of Trp28 (as well as interacting with the backbone of Asn167), thereby contributing to the position of the core scaffold of all presented carbapenem substrates. Due to the multiple tail conformations in imipenem, the orientation for the π-sulfur interaction in this case is not always optimal; however, the distance remains consistent. In addition, Trp28 shows interactions with the pyrroline methyl group (C-21), present in all the antibiotics studied other than imipenem. These interactions lead to restricted motion in the side chain of Trp28, which further rigidifies the L1 loop (Fig. 3). The lack of this interaction in imipenem could lead to a reduction in binding interactions to the loop and may contribute to the observed reduction in affinity (increase in *K_m_*) for imipenem (Table S2). It is likely that there is a hydrophobic interaction between the ring itself and the tryptophan in the absence of this methyl. Two other hydrophobic L1 residues, Val25 and Val31, also form alkyl interactions with C-21, where present.

### Binding modes of the antibiotic tails to IMP-13.

Analysis of the interactions between the carbapenem tail moieties and the surrounding residues and solvent molecules shows a complex network of position-dependent contacts. Due to the presence of more than one molecule in the unit cell, a representative molecule for which the crystal packing does not affect the antibiotic tail placement was chosen for discussion. Higher solvent accessibility of the tails leads to less restrained positions, characterized by increased B-factor values.

The most solvent-exposed parts of the four antibiotics are very distinct, while in addition, imipenem has no pyrrolidine ring in the antibiotic tail. The pyrrolidine ring of meropenem, doripenem, and ertapenem is suitably located to form aromatic, π-alkyl interactions with His197 and is further stabilized by the hydrophobic environment created by Val25 and Val31. The terminal nitromethyl group of meropenem is stabilized by direct as well as water-mediated interactions with the backbone of His163 and Gly164 and π-alkyl interactions with Trp28. The sulfonamide moiety of doripenem forms several hydrogen bonds: the nitrogen of the primary amine group interacts with Thr32 and the backbone of Val30, while the oxygen creates hydrogen bonds with backbone and side chain atoms of Thr32. The terminal part of the imipenem tail was modeled in three different conformations (50%:25%:25% occupancy), highlighting the extreme flexibility of this moiety. Due to the different conformers, the interaction network is different in every modeled position: conformer A creates a hydrogen bond with the backbone carbonyl of Val30 and water-mediated hydrogen bonds with the backbone of Thr32. The most solvent-exposed conformation, conformer B, lacks interactions with surrounding residues, most likely interacting with a water network, while conformer C interacts with the wider water network. In each case, a water molecule replaces the amine group from the other two conformer positions.

### NMR measurements show altered dynamics in the presence and absence of antibiotic.

As discussed above, the L1 loop adopts very different conformations in the two apo structures (apo_closed_ and apo_open_). In the apo_closed_ structure, the loop is folded over the active site, while in the apo_open_ structure, L1 is extended away from the protein, leaving the active site accessible. In complex with each of the hydrolyzed carbapenems, this loop forms a tightly locked, tunnel-like structure around the hydrolyzed antibiotic (Fig. 1D), with several hydrophobic interactions appearing to stabilize this state.

To further understand the role of the L1 loop in antibiotic binding, NMR spectra were acquired for IMP-13 in the apo state and for IMP-13 bound to ertapenem. An overlay of ^1^H,^15^N heteronuclear single quantum coherence (HSQC) spectra for the two forms (Fig. SI7) shows substantial chemical shift changes on addition of ertapenem, necessitating backbone assignment using triple resonance spectra in both forms. ^1^H,^15^N assignments from a total of 219 residues (excluding the 9 prolines) were achieved for 203 residues in the apo form (93%) and 195 residues in the ertapenem-bound form (89%). The assignments are shown in Fig. SI8 for the apo state and Fig. SI9 for the ertapenem-bound state.

**FIG 4:**
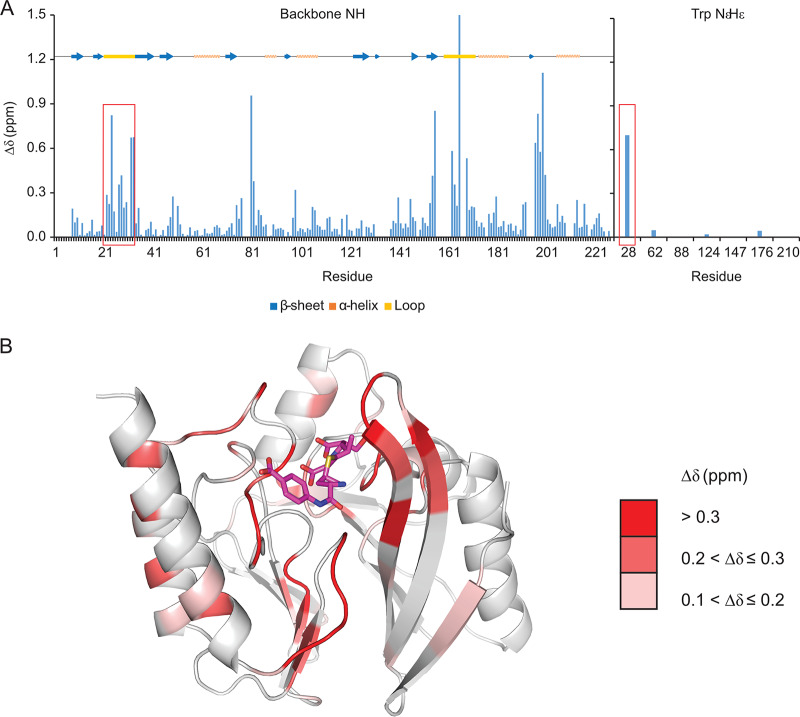
Chemical shift changes between the apo and ertapenem-bound forms of IMP-13. (A) ^1^H,^15^N backbone amide and tryptophan amide side chain chemical shift changes between the apo and ertapenem-bound forms of IMP-13 for the spectra shown in Fig. SI7 in the supplemental material (shown individually in Fig. SI8 and SI9) plotted against the residue number. A representation of the secondary structure of IMP-13 is shown above the plot. The L1 region is indicated by red boxes. (B) The shift changes (Δδ > 0.1) identified in panel A are shown on the ertapenem-bound crystal structure of IMP-13 (PDB accession number 6RZS).

Chemical shift perturbations are shown in Fig. 4 and plotted on the ertapenem-bound structure (PDB accession numbers 6RZS). The largest changes are colored in red on the structure and predominantly localize to loops in the vicinity of the ertapenem-binding pocket. These changes are also marked with arrows on the spectra shown in Fig. SI7. As expected, significant shifts were seen for residues in the L1 loop (marked with red boxes in Fig. 4), in particular, residues Glu24, Gly27, Trp28, Thr32, and Lys33, as well as the side chain NεHε of Trp28; the L3 loop (residues 163 to 166, especially residue Gly166), which lies in the vicinity of the ertapenem tail; and residue Asp81, which coordinates Zn2, as well as in the linker between B11 and A5 (residues 197 to 201). Smaller changes were seen in the β-strands B9, B10, and B11. These changes are consistent with the observations in the crystal structure (see above).

**FIG 5:**
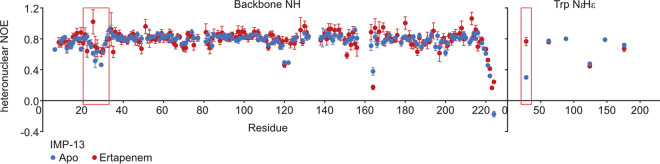
Heteronuclear NOE data showing fast-timescale motions of apo and ertapenem-bound IMP-13. Heteronuclear NOE data for the backbone amides and tryptophan indole NεHε measured at 600 MHz (^1^H frequency) and 25°C for both the apo and ertapenem-bound forms. The loop 1 region is marked with a red rectangle. Residues above residue 224 were removed from the plot, as all showed negative heteronuclear NOEs in both the apo and ertapenem-bound forms.

Heteronuclear ^1^H-^15^N nuclear Overhauser effect (hetNOE) experiments were acquired to detect fast (picosecond-nanosecond)-timescale motions (52, 53). Typically, structured regions of the protein show hetNOE values of >0.8, while flexible loops and the N and C termini show lower values (<0.8). Figure 5 shows an overlay of the hetNOE values for the apo and ertapenem-bound forms. Both states show similar values, with an average hetNOE value, taken across backbone residues Asp6 to Glu219, of 0.783 (standard deviation, 0.078) for the apo form and 0.799 (standard deviation, 0.096) for the ertapenem-bound form. However, notably lower values were recorded in the L1 loop in the apo state, with values of 0.51 and 0.46 for residues Asn26 and Gly29, respectively, whereas in the ertapenem-bound state, the values did not drop below 0.6 in this region. Most significantly, Trp28 NεHε had a hetNOE of 0.3 in the apo form, which rose to 0.77 in the ertapenem-bound state, comparable to the values for the backbone amides in structured regions of the protein, indicating a significant change in dynamic properties. This suggests that in the apo form the L1 loop is undergoing fast-timescale motions, while binding of antibiotic in the active site stabilizes the L1 loop. The restricted motion of Trp28 NεHε suggests that the antibiotic interacts with this residue, reducing the fast-timescale motions at this position. The hetNOE data show slightly more restriction in residues 165 to 168 in the presence of ertapenem, but residue Gly164 is considerably more flexible in both the apo and the ertapenem-bound states. It was not possible to assign residues at the beginning of the L3 loop, suggesting unfavorable dynamics in this region.

### Molecular dynamics simulations show significant variations in L1 loop dynamics between complex structures.

The distinct conformations observed in the two apo crystal structures and the NMR relaxation data indicate that the L1 loop is likely flexible in solution. We therefore performed molecular dynamics (MD) simulations to assess the movement and flexibility of this protein fragment on the nanosecond timescale for the apo and carbapenem-bound structures (Fig. 3). During a total simulation time of 50 ns for each system, none of the systems showed significant large-scale fluctuations, indicating that the solute systems were stable. In addition, the ligand RMSD values (Table S3) suggest that the conformation of the hydrolyzed ligands remains stable during both the 50-ns and 100-ns simulation times. Torsion angles were also generally maintained throughout both the simulation runs. Excluding the intrinsic flexibility of L1, the protein overall does not undergo significant conformational changes, aside from those in the active-site region.

However, the simulations reveal significant changes in L1 behavior between the different structures. The lower root mean squared fluctuations (RMSFs) for heavy atoms of L1 residues in the simulated doripenem-, ertapenem-, and meropenem-bound complex structures than for those of the apo_closed_ structure indicate that L1 is more rigid in the closed conformation when these ligands are bound, while in the apo protein, L1 can move with a higher degree of freedom. This is shown by the C-α RMSF being 1.4, 2.1, and 1.9 Å larger in the apo structure than in the doripenem-, ertapenem-, and meropenem-bound forms, respectively (1.1, 1.4, and 0.8 Å, respectively, for the replicas). However, while the behavior of L1 is comparable for the doripenem-, ertapenem-, and meropenem-bound structures and shows the greatest restriction for these structures, it differs significantly for the imipenem complex, which is comparable to that for the apo form (Fig. 3C and E). In the X-ray structures, the interaction of bound ertapenem and meropenem with the Trp28 side chain occurs via the sulfur adjacent to the β-lactam ring and, additionally, via the methyl group on the pyrroline ring. In contrast, imipenem lacks this additional methyl group, which reduces the strength of the lipophilic interaction with the Trp28 side chain, thus resulting in greater loop flexibility. Furthermore, as shown by both shorter and longer overall simulation times for all the complexes, the tail of imipenem shows high flexibility and occupies multiple rotamer positions; thus, it does not contribute further to the loop stability. Doripenem, however, does contain both a sulfur-π interaction and a methyl-π interaction, but the MD simulation shows a higher motility of the sulfonamide tail of the hydrolyzed antibiotic (atoms N-18, N-19, O-21, O-22, and S-28; Fig. 3A). This is in agreement with the chemical character of this moiety, which, due to a higher energy contribution to desolvation, is more prone to interact with nearby solvent molecules, thus leading to a markedly higher RMSF for the atoms in the antibiotic tail in comparison to the RMSFs for the ertapenem and meropenem structures.

## DISCUSSION

We present the apo and complex structures of IMP-13 bound to hydrolyzed carbapenems. Imipenemases represent one of the major groups of class B1 metallo-β-lactamases found in Gram-negative pathogens, and these enzymes can hydrolyze all bicyclic β-lactam antibiotics. This includes carbapenems, which are often reserved for use as the treatment of last resort in cases of multidrug resistance. There are relatively few structures of imipenemase enzymes available, and no structures of imipenemase enzymes bound to carbapenems are available. Currently, no inhibitors of metallo-β-lactamases are available in the clinic. Consequently, understanding the structural features of carbapenem interactions with a member of the imipenemase class is essential to developing new inhibitors to treat infections caused by multidrug-resistant pathogens.

Our data demonstrate that the key interactions in the bound structures are found between the conserved carbapenem core (the β-lactam and pyrrole rings and the exocyclic sulfur) of the antibiotic and the divalent zinc ions, as well as the backbone and side chain residues of the IMP-13 active site, particularly the L1 and L3 loops. Notably fewer interactions are made with the antibiotic tail region, leading to high flexibility, which likely affects enzyme efficiency, rendering careful design of the tail section key in drug discovery efforts. That IMP-13 is not selective toward the antibiotic tail region likely contributes to its broad-spectrum activity, which makes the B1 class metallo-β-lactamases particularly challenging resistance determinants. A key feature of the binding mode is the interaction between the tryptophan of L1 and the carbapenem scaffold: the tryptophan forms a closed tunnel over the β-lactam ring, thus locking the loop and the antibiotic in place (Fig. 1D). In the apo-state crystal structures, two positions for the L1 loop, open and closed, were observed. Molecular dynamics simulations also showed different degrees of flexibility in the L1 loop region. These results are supported by the fast-timescale motion for loop L1 observed in the apo form in the hetNOE experiment, which was reduced in the presence of the antibiotic ertapenem (Fig. 5), and, in particular, the substantial reduction in the flexibility of Trp28HεNε between the apo and ertapenem-bound states. These observations are consistent with the results of previous NMR studies on a subclass B1 dizinc metallo β-lactamase from Bacteroides fragilis (54–56), where L1 loop residues show lower hetNOE values in the free form than in the presence of a tightly binding inhibitor, most notably, for the L1 tryptophan indole (Trp28 for IMP-13, Trp49 [56]), indicating a potentially important role of the L1 loop in substrate recruitment and stabilization during the hydrolysis reaction. Previous studies have discussed whether the tryptophan and other hydrophobic residues in the L1 loop may act as a recruiter, loosely binding the substrate in the open formation and then moving to the closed formation to aid substrate addition to the binding site (54, 56).

Nevertheless, it is clear from the MD simulations (Fig. 3) that differences in antibiotic structure affect the restriction of the L1 loop, with the doripenem, ertapenem, and meropenem complexes showing the greatest restriction in L1 loop mobility. This is consistent with the kinetic parameters reported for IMP-13 (see Table S2 in the supplemental material), showing tight binding for meropenem and ertapenem (*K_m_* values, in the low-micromolar and high-nanomolar ranges, respectively). In contrast, imipenem shows weaker binding (*K_m_*, ca. 50 μM), consistent with the higher L1 flexibility. Notably, the *k*_cat_ for imipenem is 2 orders of magnitude higher than that for meropenem and ertapenem. Given that product release, preceded by the necessary L1 opening, likely determines the turnover rate, this indicates that tighter binding reduces the turnover rate of IMP-13. Consequently, an efficient, noncovalent inhibitor could interact with and stabilize the L1 loop in the closed conformation, forming a principle for inhibitor design.

**FIG 6:**
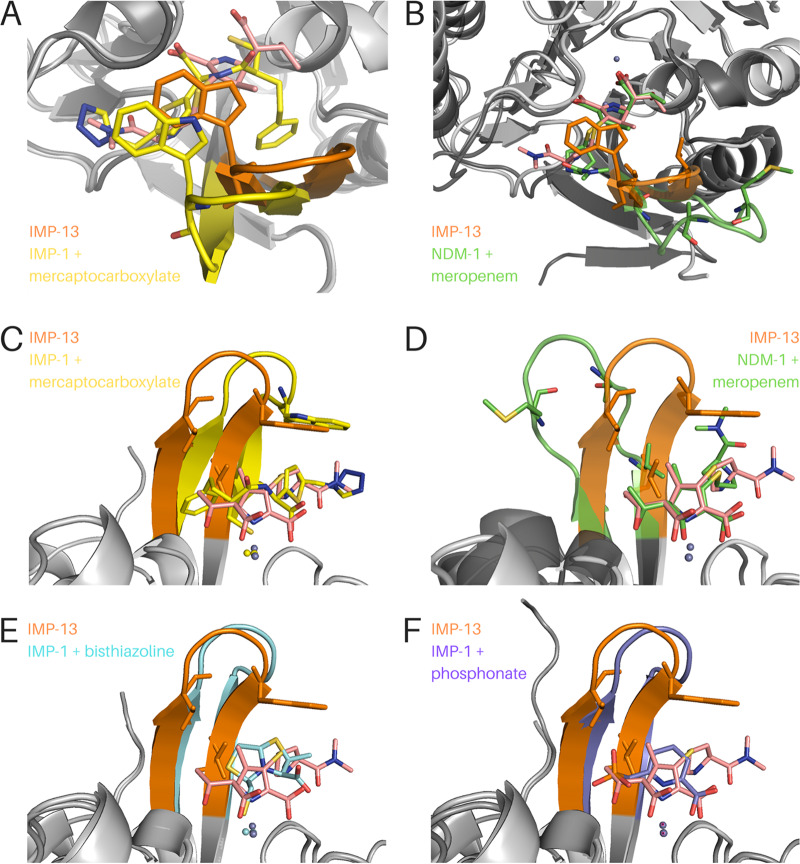
Comparison of the IMP-13 meropenem-bound structure with previously published metallo-β-lactamase structures. IMP-13 is always depicted with an orange loop, and the IMP-13-bound meropenem is depicted in salmon. (A and C) Two views of IMP-13 overlaid with IMP-1 bound to the mercaptocarboxylate inhibitor (yellow; PDB accession number 1DD6 [60]). (B and D) Overlay of IMP-13 with NDM-1 and hydrolyzed meropenem (green; PDB accession number 5N0H [67]). The phenylalanine residue at the tip of the loop is not resolved in the structure, but the backbone is shown as sticks. (E) Overlay of IMP-13 with IMP-1 bound to the bisthiazoline inhibitor L-VC26 (cyan; PDB accession number 5EWA [57]). (F) Overlay of IMP-13 with IMP-1 bound to a phosphonate-based inhibitor (purple; PDB accession number 5HH4 [61]).

IMP-13 shows 83% and 92% sequence identity with the IMP-1 and IMP-2 forms, respectively, and is quite divergent from other variants (41). Consequently, it is instructive to compare our structures to those of other available MBL structures (Fig. 6; Fig. SI10).

Comparison of IMP-1 structures (PDB accession numbers 5Y5B and 5EV6 [57]) with both our apo_closed_ and carbapenem-bound IMP-13 structures yields differences in the L1 region. The amino acid sequences are highly conserved between the L1 regions of the two structures, with only one difference being seen, which is at the C-terminal end of the loop (Pro32 in IMP-1 is changed to Thr in IMP-13). In one of the IMP-1 structures, the β-strand of the loop at this point is seen to be straighter and farther out from the active site than in the case of the structures that we have presented, despite the tip of the loop being closer to the active site than in the open-conformation IMP-13 structure presented here. It is likely that the Pro32-to-Thr mutation leads to a more flexible loop in IMP-13 and a more restricted loop in IMP-1, as a result of the more constrained dihedral angles of proline. Mutagenesis analysis of IMP-18 (58) (a subclass B4 β-lactamase [31]) also indicates that this residue has a key effect, as the turnover rates of the enzyme are significantly altered (3- and 10-fold increases in the *k*_cat_ for imipenem and meropenem, respectively) on mutation from threonine to proline. IMP-2 also lacks Pro32 at the end of L1, which is instead mutated to Ser. The IMP-2 structure (PDB accession number 4UBQ [59]) shows that the loop is found between the locations of the loop in the apo_open_ and apo_closed_ structures reported here. The varying extent of the β-strand structure (subclasses B2 and B3) on either side of the L1 loop may reflect the dynamic nature of this region, and thus, the loop can be captured in different conformations in different crystal structures.

The ligands crystallized previously with IMP-1 belong to different compound classes, and hence, we compared those structures with our carbapenem-bound structures to identify whether similar interactions are exploited. Comparison with the IMP-1 structure bound to the mercaptocarboxylate inhibitor (60) (PDB accession number 1DD6) shows that the benzyl ring is pointing toward the loop (residues 21 to 23). Were the loop in the same position as in IMP-13, this would clash with the loop position, particularly with Val31. The position of the loop in IMP-1 is shifted laterally by approximately 1 Å. This could indicate potential binding selectivity to IMP-1 or an alternative explanation of sterically induced loop movement. The free thiol of mercaptocarboxylate is coordinated by the two zinc atoms, displacing the nucleophilic water, equivalent to the carboxylate of β-lactams. In contrast, both the tertiary amine and the sulfur of the thiophene ring point in an orientation opposite to that seen in comparable antibiotic residues. This suggests that further structural optimization, based on a knowledge of antibiotic binding, could be used to optimize inhibitor interactions.

The structures of bisthiazolidine inhibitors bound to IMP-1 (PDB accession number 5EWA) (57) show a number of interactions mimicking those of antibiotic binding. The free thiol is coordinated by Zn1 and Zn2, displacing the nucleophilic water, while the thiazoline rings interact with the L1 tryptophan, creating stacking interactions. The carboxylate interacts with the lysine residue in L3, equivalent to the β-lactam carboxylate. In contrast, a phosphonate inhibitor (61) (PDB accession number 5HH4) does not displace the nucleophilic water, with the phosphonate group coordinating Ser119 (IMP-1 numbering) and the nucleophilic water. The pyridine nitrogen and carboxylate interact with Zn2 and the L3 lysine, again making interactions similar to those observed in our antibiotic-bound structures. The pyridine ring makes a T-shaped π-stacking interaction with the L1 tryptophan. These comparisons suggest that mimicking the key interactions found in the antibiotic complex structures presented in this paper is important in designing inhibitors.

Comparing our IMP-13 structures to the structure of the NDM-1 β-lactamase, another broad-spectrum MBL of clinical relevance, the key difference is the replacement of the tryptophan of L1 (Trp28) in IMP-13 with a phenylalanine in all 17 NDM variants (62). Consequently, this suggests an alternative mode of binding. In the published structure of NDM-1 in complex with hydrolyzed meropenem (PDB accession number 4EYL, which was rerefined with PDB accession number 5N0H) (63), the loop is shown in the open conformation and therefore does not form a closed tunnel covering the β-lactam ring. In contrast, Trp28 of IMP-13 shows direct interactions with the bound carbapenems, while the equivalent Phe70 of NDM-1 is more than 7 Å away and does not interact with the ligand. The conservation of this residue in all known NDM-1 variants indicates the importance of this amino acid for the proteins’ activity. The residue equivalent to Val25 (Met in NDM-1), which in IMP-13 flanks the flexible loop section and interacts with C-21 of meropenem, is seen in NDM-1 in a location where this interaction is removed altogether. The equivalent of Val31, the other flanking valine, is also seen in an altered position. This is farther away from C-21 of the antibiotic but is closer to the methylamine group, which could explain why this group of the meropenem molecule is in a different location in this structure, differing in position by about 5 Å.

We extended our comparison to structures of a variety of NDM proteins bound to various ligands that were deposited in the Protein Data Bank (PDB) in 2017 and 2018 (PDB accession numbers 4TYF, 4TZ9, 4TZB, 4TZE, 4TZF, and 5WIG [64], 5WIH [64], 5XP9 [65], 5A5Z [66], 5N0H [67], 5N0I and 5YPK [46], 5YPL [46], 5YPN [46], 6EX7 [67], 5YPM [46], and 5JQJ, 5K4M, and 5XP6 [65]). Notably, none of these structures show a fully closed-tunnel conformation like that seen in the IMP-13–carbapenem complexes presented here. The backbone is seen in a half-closed formation in many of these PDB accessions (e.g., PDB accession numbers 5JQJ, 5K4M, and 5XP6), but the chain never fully reaches over the substrate. This could lead to a reduced contact area between the ligand and the protein. The reduced hydrophobicity of this loop in NDM-1 could also explain the higher *K_m_* (lower affinity) of meropenem and imipenem (Table S2) relative to that of the IMP enzymes (28).

**FIG 7:**
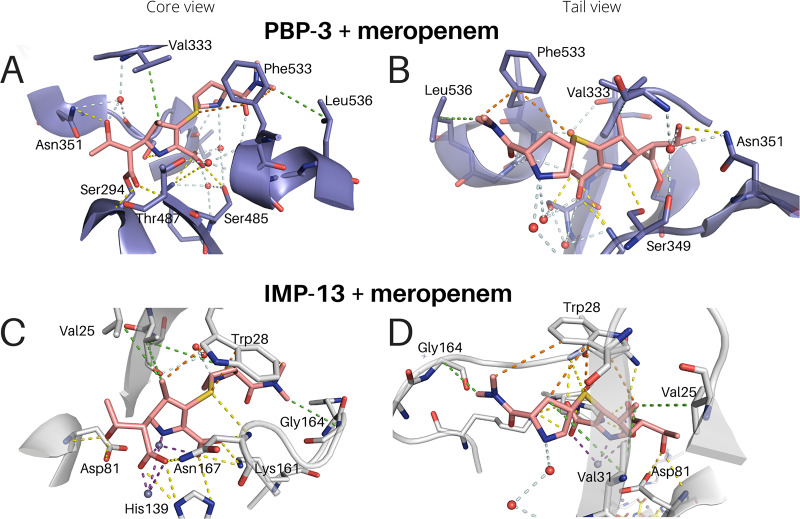
Comparison of PBP-3 (3PBR) and IMP-13 binding to meropenem. View of the carbapenem scaffold (A and C) and of the tail moieties (B and D). Interactions are shown as dashed lines. Purple, zinc interactions; yellow, H bonds and charge-charge interactions; orange, aromatic interactions; green, hydrophobic interactions; pale blue, water network.

We also compared our results to the natural target of β-lactam antibiotics, the penicillin binding proteins (PBP). A structure of penicillin binding protein 3 (PBP-3 [3PBR]) (68) bound to meropenem is available, facilitating comparison between the interactions of meropenem with a β-lactam target protein (PBP) and the enzymes (β-lactamases) that degrade it (Fig. 7). From the point of view of drug development, comparative studies could highlight key similarities and differences, aiding with the development of new antibiotics in this class with lower susceptibility to the β-lactamase-driven degradation. The major interactions, primarily hydrophobic, between the protein and the antibiotic are maintained, but key differences are observed. First, the central Trp28 interaction of IMP-13, both to the sulfur and to C-21 of the core carbapenem scaffold, is replaced by a hydrophobic interaction with Phe533 of PBP-3. The interactions from His139 on L3 are partially emulated in PBP-3 by Gly486, while Asn167 and Cys158 provide interactions similar to those provided by Thr487 and Lys484 of PBP-3, respectively. However, while these interactions are different, they are very similar in character. It is also interesting to note the difference between the meropenem and imipenem interactions. In PBP, the imipenem scaffold (lacking C-21) interacts with Tyr532, while C-21 of meropenem displaces this and Phe533 rotates 180° around the chain to form an alternative hydrophobic environment in the vicinity. Despite the strong Tyr interaction and the fact that this is closer to the native position than it is in the meropenem-bound form, imipenem binds by a factor of 16 times weaker than meropenem (68), indicating that the tail plays a much stronger role in the binding to PBP than to IMP-13, where the carbapenem scaffold and alterations to it are more important. PBP-3 binding also utilizes an intricate water network to align for formation of the covalent bond with Ser294, which ultimately deactivates the protein. Neither covalent interactions nor a water network is seen in the IMP-13 or NDM-1 structure. The similarities at play highlight the challenge that drug discovery programs face in this area, but the differences may provide opportunities that can be exploited to deliver novel pharmaceutical solutions.

We report high-resolution structures of IMP-13 in diverse functional states and with different ligands bound. The structures explain the specificity of the enzymatic mechanism and the molecular recognition of substrates by the IMP-13 β-lactamase. The data presented and the comparisons presented above suggest that in IMP-13 the active-site loop plays a central role in antibiotic binding, with the primary interactions being to the core carbapenem scaffold. Consequently, such identified motifs that cause restriction in the L1 loop flexibility could form important parts of β-lactamase inhibitors engaging and stabilizing the loop in the closed conformation and blocking access of the natural substrates. The substantial chemical shift changes observed by NMR in the tryptophan indole region (Fig. 4; Fig. SI7) could be used in high-throughput screening to identify ligands with the potential to stabilize the active-site loop in the closed conformation, similar to previous two-dimensional screening approaches focusing on spectral properties specific to the system of interest (69).

On the other hand, the ability of the loop to adopt a fully open state, as observed in the apo_open_ structure of IMP-13, provides an alternative strategy for inhibitor development. Most drug development strategies based on these targets have so far been aimed at the active site itself. As the loop appears to play an active role in the binding of ligands to the protein, prevention of the loop closing by an allosteric inhibitor could have a similar inhibitory effect.

The presented crystal structures and experimental NMR data combined with our molecular dynamics simulations provide complementary information about changes in conformational dynamics linked to ligand binding that should be considered in the development of small-molecule inhibitors.

## MATERIALS AND METHODS

### Protein expression and purification.

The mature forms of the IMP-1 (residues 29 to 276) and IMP-13 (residues 21 to 246) proteins without a signal peptide were cloned into a pET-SUK vector (70). The constructs were transformed into Escherichia coli BL21(DE3) cells and plated on LB agar supplemented with kanamycin (50 μg/ml). The cells were grown in ZYM 5052 autoinduction medium (71) at 37°C until the optical density at 600 nm (OD_600_) was 2.0, and thereafter, the protein was expressed at 20°C overnight. The cells were collected by centrifugation, resuspended in lysis buffer [50 mM Tris-HCl, pH 8.0, 300 mM NaCl, 5 mM β-mercaptoethanol, 20 mM imidazole supplemented with 4-(2-aminoethyl)benzenesulfonyl fluoride hydrochloride (AEBSF), DNase I, lysozyme], and lysed by sonication. The lysate was clarified by centrifuging for 45 min at 27,000 rpm, and the pH was adjusted to 8.0. The resulting supernatant was then passed twice over a HisTrap Excel column (GE Healthcare) that had been preequilibrated with lysis buffer. The column was washed with binding buffer (50 mM Tris-HCl, pH 8.0, 300 mM NaCl, 5 mM β-mercaptoethanol, 20 mM imidazole, 10 mM ZnCl_2_), and protein was eluted with elution buffer (50 mM Tris-HCl, pH 8.0, 300 mM NaCl, 5 mM β-mercaptoethanol, 300 mM imidazole). SUMO hydrolase (dtUD1) (72), provided by the Protein Expression and Purification Facility (PEPF; Helmholtz Zentrum München), was added to the eluted protein, and the components were gently mixed and incubated for 1 h at room temperature, followed by buffer exchange to binding buffer. A second step of affinity chromatography was performed to remove the SUMO tag and SUMO protease. The IMP-1/13-containing fractions were then concentrated and purified to homogeneity using a Superdex 75 size exclusion column preequilibrated with 5 mM Tris, pH 8.0, 50 mM NaCl, 5 mM β-mercaptoethanol, and 10 μM ZnCl_2_.

For isotopically labeled expression, cells were grown in M9 minimal medium supplemented with ^15^NH_4_Cl and [^13^C]glucose and induced as described above at an OD_600_ of 1, with overnight expression at 20°C. Cells were collected by centrifugation and resuspended in lysis buffer (100 mM Tris, 300 mM NaCl, 5 mM β-mercaptoethanol, pH 8.0) supplemented with DNase I and AEBSF. The supernatant was passed twice over Zn-nitrilotriacetic acid (NTA) beads. In our hands, NDM-1 was observed (as seen by paramagnetic effects in the NMR spectra) to bind Ni from the column in its active site; therefore, to prevent the possibility of the same occurring with IMP-13 and affecting the spectral quality, the NTA beads were loaded with Zn to ensure that the IMP-13 metal binding site was loaded with Zn. The column was preequilibrated with lysis buffer as described above, and SUMO hydrolase was added to the protein on the column and left overnight at room temperature. The cleaved IMP-13 was eluted with 5 ml lysis buffer and further purified using Superdex 75 size exclusion chromatography with a column that had been preequilibrated with NMR buffer (50 mM HEPES, 100 mM NaCl, pH 7.0). Samples were supplemented with 10% D_2_O for NMR spectroscopy.

### Crystallization of IMP-13 in apo and carbapenem-bound forms.

Purified protein was concentrated to 12 mg/ml, and screening for crystallization conditions was performed using commercially available buffer sets in a sitting-drop vapor diffusion setup by mixing 0.2 μl of protein complex solution and 0.2 μl of buffer solution. For cocrystallization, meropenem or doripenem powder was added to the protein solution (final concentrations, 100 and 50 mM, respectively) and incubated for 30 min. For cocrystallization of IMP-13 with ertapenem and imipenem, antibiotic powder was dissolved in crystallization buffer and mixed with protein to a final concentration of 5 and 25 mM, respectively. All crystals were obtained at room temperature from solutions containing 0.1 M Tris, pH 8.5, 25% polyethylene glycol (PEG) 4000 (apo_open_, in which the loop is open; PDB accession number 6R79), 0.1 M sucrose-phosphate-glutamic acid buffer, pH 8.0, 25% PEG 1500 (apo_closed_, in which the loop is closed; PDB accession number 6R78), 0.1 M bis-Tris, pH 6.5, 25% PEG 3350 (meropenem complex; PDB accession number 6R73), 0.1 M bis-Tris, pH 5.5, 0.2 M ammonium sulfate, 25% PEG 3350 (imipenem complex; PDB accession number 6RZR), 0.1 M trisodium acetate, pH 5.6, 0.2 M ammonium acetate, 30% PEG 4000 (ertapenem complex; PDB accession number 6RZS), and 0.1 M sodium HEPES, pH 7.5, 25% PEG 2000 monomethyl ether (doripenem complex; PDB accession number 6S0H).

### Structure determination and refinement.

Crystals were cryoprotected in 20% glycerol (apo), 2-methyl-2,4-pentanediol (meropenem complex), or 25% ethylene glycol (imipenem, ertapenem, and doripenem complexes) in the mother liquor and flash-frozen in liquid nitrogen. The diffraction data were collected at the id30b beamline at ESRF (Grenoble, France) and on the X06DA beamline at the Swiss Light Source (Paul Scherrer Institut, Villigen, Switzerland). The data were indexed and integrated using the XDS program package (73, 74) and scaled and merged using the Aimless program (75). The initial phases were obtained by molecular replacement, calculated using Phaser software (76) and the IMP-1 structure as a search model (PDB accession number 1DD6 [60]). The initial model was manually rebuilt according to the resulting electron density maps using the Coot program (77). The structures of IMP-13 in complex with hydrolyzed carbapenems were solved using the same approach and the IMP-13 apo structure as a search model. Carbapenem geometrical restraint files were created using the Grade web server (78). Restrained refinement was performed using the Phenix or Refmac program, with additional restraints being generated using the proSMART program (79–81). Five percent of the reflections were used for cross-validation analysis, and *R*_free_ was employed to monitor the refinement strategy. Water molecules were added using the Coot program and afterwards were manually inspected. The final models were deposited in the Protein Data Bank under accession numbers 6R79 and 6R78 for the apo forms and 6R73, 6RZR, 6RZS, and 6S0H for the meropenem, imipenem, ertapenem, and doripenem bound forms, respectively. Interactions were visualized with Biovia Discovery Studio Visualizer software (82). All molecular graphics were prepared using the PyMOL (83) or Maestro (84) program. Crystallographic parameters are shown in Table S4 in the supplemental material.

### NMR spectroscopy.

NMR experiments were recorded at 298 K on Bruker Avance III 600-MHz and 800-MHz spectrometers (^1^H frequency; 600 or 800 MHz, respectively) equipped with a 5-mm TCI or QCI cryoprobe. For assignments, ^1^H,^15^N HSQC, three-dimensional (3D) ^15^N-edited nuclear Overhauser effect spectroscopy (NOESY), HNCA, HN(CO)CA, HNCACB, HN(CO)CACB, and CBCACONH experiments were recorded on uniformly ^15^N,^13^C-labeled samples. Samples at 0.5 to 0.6 mM were prepared in NMR buffer (50 mM HEPES, 100 mM NaCl, pH 7.0) supplemented with 10% D_2_O. For the ertapenem assignment, the sample was supplemented with 5.7 mM ertapenem (a ca. 10-fold excess). Spectra were recorded with 2.9 mM and 5.7 mM ertapenem added, and with no further changes being observed between the two spectra, the protein was assumed to be saturated. Backbone assignment experiments (except for the 3D NOESY experiments) were recorded with 25% nonuniform sampling, using Poisson-gap sampling (85), and reconstructed using the Cambridge CS package and the CS-IHT algorithm (86). Heteronuclear NOE experiments were recorded at 600 MHz and 298 K using a sequence with interleaved saturated and unsaturated planes (53). Spectra were acquired with 2,048 by 300 complex points and a recycle delay of 1.2 s with 32 scans. NOEs were calculated as the ratio of the results of saturated to unsaturated experiments. Errors were calculated using the standard deviation of the noise. All spectra were processed with zero filling and Gaussian and/or sinebell window functions in the direct dimension and a sinebell window function in the indirect dimension. The water signal was removed by convolution with a sine function. The spectra were processed in the Azara program (W. Boucher, unpublished data) and analyzed using CcpNmr Analysis (87). Chemical shift perturbations (Δδ values) were calculated according to the following formula:

$$\text{Δδ}=\sqrt{{\left({\text{Δδ}}_{\text{HN}}\right)}^{2}+{\left({\text{Δδ}}_{15_{\text{N}}}\right)}^{2}/6}$$

### Antibiotic hydrolysis assay.

Enzymatic studies were carried out on the expressed proteins to confirm that the protein was in its active state. The enzymatic activity of the recombinantly produced metallo-β-lactamases was monitored as previously described (88) at 37°C in 75 mM HEPES buffer at pH 7.3, using 1 to 500 μM meropenem or imipenem as the substrate. β-Lactam hydrolysis was followed at 300 nm (Table S2).

### Molecular dynamics simulations.

Molecular dynamics simulations were run using the Maestro Desmond molecular dynamics package (version 2017.3) (89, 90). The PDB accessions of the apo_closed_ and complex structures were prepared by adding missing side chains and hydrogens using the YASARA Structure’s built-in clean command (91). The structures were then imported into the Schrödinger Maestro (version 2017.3) program and further refined using the Maestro (version 11.1) protein preparation wizard (92). Protonation states were calculated using the PROPKA program (93, 94) at pH 7.0 ± 2.0, and minimization of hydrogen positions with a restrained backbone was performed using the OPLS3 force field (95) in order to optimize the hydrogen bonding network. Both the apo_closed_ and complex systems were then prepared for simulation using the Maestro (version 11.1) system builder graphical user interface (GUI) and the TIP4P (96) solvent model (crystallographic water molecules were deleted) in an automatically generated cubic cell with periodic boundary conditions. In addition to the solvated complex, Na^+^ and Cl^−^ ions corresponding to those in a 150 mM buffer were placed in the cell in order to set the total net charge to zero. The coordination of the zinc metal centers was maintained by adding pseudo-bonds between the metals and the coordinating residues, using the default parameters for angles and charges of the OPLS-AA 2005 force field for the sake of the speed of the calculations. In the case of the apo protein, a tetrahedral coordination was chosen for both the zinc atoms in the active site. Furthermore, in the apo structure, no pseudo-bond was added to the bridging water molecule observed in the crystallographic structure, thus reducing the coordination of the metal centers to three residues to simulate the hydration sphere around the zinc atoms. For all the other complexes, the geometry of the zinc bound to histidines was considered tetrahedral (with a coordination number of 4) and that of the other zinc atom was considered to be octahedral (with a coordination number of 6).

Simulations of the systems were run using the Maestro Desmond molecular dynamics GUI for a total simulation time of 50 ns to ensure system convergence (this was checked on the RMSD plot of the simulations), recording at intervals of every 50 ps (1,000 snapshots in total) for *xyz* coordinates and 1.2 ps for potential energy calculations of the ensemble. Replicates of the simulations were performed using the Desmond molecular dynamics package (version 2019.3) in Maestro (version 11.8). One replica for each system was simulated for 100 ns, recording at intervals of every 100 ps (1,000 snapshots in total) for *xyz* coordinates and 2.5 ps for potential energy calculations. Both for the first run of production MD and for the replicates, the ensembles were set to a constant temperature (300 K) and pressure (1.01 × 10^5^ Pa); the force cutoff radius was set to 9.0 Å, and each solvated model was relaxed with the Desmond default relaxation protocol before starting the simulation. Simulations were performed on a standard personal computer workstation (Intel Core i7 5960x, 32 GB random-access memory) using an Nvidia GeForce 1070 graphics processing unit.

### Accession number(s).

Coordinates and structure factors have been deposited in the Protein Data Bank under accession numbers 6R79 for the apo loop open form, 6R78 for the apo loop closed form, 6R73 for the meropenem complex, 6RZR for the imipenem complex, 6RZS for the ertapenem complex, and 6S0H for the doripenem complex. NMR assignments have been deposited in the BMRB under accession numbers 50012 for the apo form and 50013 for the ertapenem-bound form.

## Supplementary Material

Supplemental file 1
