# Supplementary material for: Structure and Molecular Recognition Mechanism of IMP-13 Metallo-β-Lactamase
Source: Antimicrob Agents Chemother. 2020 May 21;64(6):e00123-20. doi: 10.1128/AAC.00123-20 (PMC7269475; doi:10.1128/AAC.00123-20)
Supplement: Supplemental file 1 [file AAC.00123-20-s0001.pdf]

Supplemental Information

Structure and molecular recognition mechanism of IMP-13 metallo-β-lactamase

Charlotte A. Softley[a,b,1], Krzysztof M. Zak[b,1], Mark J. Bostock[a,b,1], Roberto Fino[a,b], Richard Xu Zhou[a,b], Marta Kolonko[b,c], Ramona Mejdi-Nitiu[d], Hannelore Meyer[d], Michael Sattler[a,b,*], Grzegorz M. Popowicz[a,b,*]

[a] Biomolecular NMR and Center for Integrated Protein Science Munich at Department Chemie, Technical University of Munich, Lichtenbergstraße 4, 85747, Garching, Germany.

[b] Institute of Structural Biology, Helmholtz Zentrum München, Ingolstädter Landstraße 1, 85764 Neuherberg, Germany.

[c] Department of Biochemistry, Faculty of Chemistry, Wroclaw University of Science and Technology, Wybrzeze Wyspianskiego 27, 50-370, Wroclaw, Poland

[d] Institute for Medical Microbiology, Immunology and Hygiene, Technical University of Munich, Trogerstraße 30, 81675 München, Germany

* corresponding author emails:

grzegorz.popowicz@helmholtz-muenchen.de; michael.sattler@helmholtz-muenchen.de;

[1] **These authors contributed equally to this work.**

Contents

SI Table 1. Interactions and Interaction Distance Summary. 3

SI Table 2. Enzymatic constants for IMP-1, IMP-13 and NDM-1. 4

SI Table 3. Selected values calculated from molecular dynamics simulations for IMP-13 apo and complex structures.. 5

SI Table 4. Data collection and refinement statistics 7

SI FIG 1. Structures of the tested carbapenems 11

SI FIG 2. Sequence alignment of IMP-1, IMP-2, IMP-13 and IMP-18. 12

SI FIG 3. Labelled protein sequence and hydrolyzed meropenem. 13

SI FIG 4. Annotated crystal structure (apo_{closed})... 14

SI FIG 5. Conserved co-ordination of Zn1 and Zn2 in the carbapenem-bound state. .. 14

SI FIG 6. mFo-DFc omit maps... 15

SI FIG 7. Overlay of $^{1}H,^{15}N$ HSQC spectra for apo and ertapenem-bound IMP-13. 17

SI FIG 8. $^{1}H,^{15}N$ HSQC spectrum for apo IMP-13 showing assigned residues............. 19

SI FIG 9. $^{1}H,^{15}N$ HSQC spectrum for ertapenem-bound IMP-13 showing assigned residues. 21

SI FIG 10. Loop 1 of different IMP variants.. 21

Interacting Atoms		Distance (Å)			
**Ligand**	**Protein**	**Meropenem**	**Imipenem**	**Ertapenem**	**Doripenem**
C3	W28		4.0		
N6	Zn2	2.1	2.1	2.4	2.9
	Zn1	3.9	4.0	4.3	3.7
	D81 COOH		2.9	3.1	
O8	K161 NH_2	2.7	2.9	2.8	2.9
	N167 N	3.0	2.7	3.2	2.8
O9	H139 ND1	3.7	3.0	4.3	
	H139 NE2			4.4	3.0
	K161 NH_2	2.8	2.7	2.9	2.7
	Zn2	2.7	2.4	2.4	2.5
S10	W28	3.6	4.1	3.7	3.6
	N167 N	3.7		3.8	
C21	V31 isopropyl	4.0	Not present	3.9	3.6
	V25 isopropyl	4.2		4.2	3.7
	W28	3.7		4.0	3.4
O24	D81 COOH	2.3	3.1/3.2	2.6/2.9	3.0/3.3
	D81 N	3.2	3.1	3.0	2.9
	S80 OH		3.5	4.0	3.8
O26	Zn1	2.8	2.9	2.4	2.9
	H139 NE2	3.3		3.0	
	N167 NH_2	2.8	2.9	3.4	2.8
O27	Zn2	3.4	3.1	3.1	3.1
	Zn1	1.7	1.9	1.9	2.1
	H139 NE2	3.2	3.4	3.1	
Tail atoms:					
N13	H_2O (T32 bridge)	2.9			
C15	W28	3.5			
NMe_2	W28	4.5			
	G164 CA	4.0			
NH	W28 CO		Conf A 2.6		
	H_2O		Conf B 3.2		
			Conf B 3.3		
NH_2	V30 CO		Conf A 3.1		
NH	H_2O			2.5	
Sulfonamide N	T32 OH				3.1
	V30 CO				3.3
Sulfonamide O	T32 N				2.7
	T32 OH				3.5/3.8

SI Table 1. Interactions and Interaction Distance Summary. Distances are shown between ligand and protein atoms described in the first two columns for the four antibiotics

studied. Where multiple conformations of the ligand are present, this is denoted ‘Conf X’. Where two atoms from the same functional group with different distances are observed, this is denoted: ‘3.4/3.2’ .

		**IMP-1**		**IMP-13**		**NDM-1**
		In-house	(1)	In-house	(2)	(3)
**Meropenem**	k_{cat} [s^{-1}]	71.1 ± 1.3	50 ± 5	0.2 ± 0.04	1.4 ± 0.1	12
	K_m [µM]	9.8 ± 1.3	10 ± 2	16.4 ± 1.8	10 ± 2	49
**Imipenem**	k_{cat} [s^{-1}]	165.9 ± 4.6	46 ± 3	64.7 ± 2.0	120 ± 5	20
	K_m [µM]	41.2 ± 4.7	39 ± 4	162.6 ± 12.4	49 ± 5	94
**Ertapenem**	k_{cat} [s^{-1}]				1.8 ± 0.1	
	K_m [µM]				0.8 ± 0.1	

SI Table 2. Enzymatic constants for IMP-1, IMP-13 and NDM-1. Data recorded for this publication are shown as mean ± standard error. References for previously published values are given.

	Zn-Zn Distance		Ligand RMSD		Trp28 RMSF Cα		Trp28 RMSF Side Chain	
	**50 ns MD**	**100 ns MD**	**50 ns MD**	**100 ns MD**	**50 ns MD**	**100 ns MD**	**50 ns MD**	**100 ns MD**
**Apo**	5.3 ± 0.3	3.8 ± 0.1	-	-	2.3	2.6	3.4	3.9
**Doripenem**	4.1 ± 0.1	4.1 ± 0.1	1.8 ± 0.2	2.4 ± 0.3	1.3	1.5	2.0	2.6
**Ertapenem**	4.3 ± 0.1	4.3 ± 0.1	1.1 ± 0.2	2.6 ± 0.6	1.2	1.2	1.3	1.9
**Imipenem**	4.3 ± 0.1	4.0 ± 0.1	1.8 ± 0.2	1.9 ± 0.3	2.2	1.5	3.6	2.0
**Meropenem**	4.3 ± 0.1	4.3 ± 0.1	1.9 ± 0.3	1.9 ± 0.4	1.1	1.8	1.5	2.9

SI Table 3. Selected values calculated from molecular dynamics simulations for IMP-13 apo and complex structures. Column 1: average Zn-Zn distance for all the complexes simulated. Column 2: average RMSD per ligand. Columns 3 and 4 show the RMSF for Trp28 in L1. All values are reported in Ångstrom and are calculated over the whole duration of the simulations (50 ns and 100 ns respectively, 1000 snapshots for each simulation). It should be noted that in the apo structure the higher average Zn-Zn distance can be explained as the zinc positions are not strongly constrained by the presence of a bridging moiety from the ligand. As a result, the zinc ions are free to move further apart and can accommodate a Cl^- from the solvation model. In the antibiotic-bound structures, the zinc ions coordinate the carbonyl moiety from the antibiotic scaffold, which provides a stronger restraint to retain the

Zn-Zn distance. While the 100 ns MD replicas show the same overall trend in L1 mobility as is observed for the shorter MD runs, the meropenem complex ensemble shows a higher RMSF for the Trp28 sidechain heavy atoms. This can be explained by the local solvation model, where negatively charged chlorine atoms, over longer simulation times, have a higher chance of interacting with the positively charged nitrogen of the meropenem tail. Due to steric hindrance, the sidechain is then forced to undergo a rearrangement, and is pushed slightly further away from the core of the antibiotic. This behaviour is observed only for short intervals; the sidechain quickly rearranges in order to re-establish the hydrophobic interactions with the antibiotic core after the chlorine atom returns to the bulk solution. The same behaviour is observed for the side chain of Trp28 in the 50 ns MD run of the imipenem complex.

Data collection	apo_{open}	apo_{closed}	IMP-13 meropenem complex	IMP-13 doripenem complex	IMP-13 ertapenem complex	IMP-13 imipenem complex
Space group	P 1	P 1 21 1	P 1 21 1	P 1 21 1	P 1 21 1	P 21 21 21
Cell dimensions						
a, *b*, *c* (Å)	44.76<br>64.82<br>86.54	60.79<br>73.12<br>64.21	57.98<br>72.37<br>61.19	61.43<br>72.32<br>62.93	54.69<br>48.10<br>92.57	48.21<br>90.92<br>120.81
α, β, γ (°)	107.5<br>91.0<br>94.8	90.0<br>112.6<br>90.0	90.0<br>108.4<br>90.0	90.0<br>111.7<br>90.0	90.0<br>105.7<br>90.0	90.0<br>90.0<br>90.0
Resolution range (Å)	61.55-1.90<br>(1.96-1.90)	51.94-2.21<br>(2.29-2.21)	58.04-2.30<br>(2.38-2.30)	36.16-2.84<br>(2.95-2.84)	18.77-2.19<br>(2.27-2.19)	45.46-1.90<br>(1.96-1.90)

Total reflections	112219<br>(11260)	78211<br>(8059)	40220<br>(4077)	82215<br>(7439)	78076<br>(7323)	277351<br>(26654)
Unique reflections	67023 (6850)	25528 (2538)	20856 (2130)	12023 (1188)	23333 (2252)	42307(4082)
Mean I/sigma(I)	9.58 (2.14)	13.47 (2.27)	7.62 (1.94)	11.57 (1.02)	7.88 (1.63)	11.65 (1.41)
Completeness (%)	94.65 (94.12)	98.31 (99.41)	97.01 (99.30)	98.84 (97.76)	97.35 (94.59)	99.24 (97.47)
Multiplicity	1.7 (1.7)	3.1 (3.2)	1.9 (1.9)	6.8 (6.3)	3.3 (3.3)	6.6 (6.5)
$CC_{1/2}$	0.997 (0.825)	0.998 (0.734)	0.995 (0.84)	0.998 (0.626)	0.992 (0.59)	0.998 (0.753)
Wilson B-factor (Å^2)	24.59	50.97	33.20	76.44	30.94	37.71
**Refinement**						
R-work	0.18	0.17	0.18	0.22	0.20	0.18
R-free	0.21	0.21	0.24	0.26	0.23	0.22
Number of atoms						

non-hydrogen atoms	7778	3552	3450	3541	3716	3819
macromolecules	6803	3341	3294	3418	3438	3481
ligands	74	78	58	92	72	136
solvent	901	133	98	31	206	202
RMSD						
bonds (Å)	0.014	0.014	0.008	0.013	0.013	0.014
angles (°)	1.73	1.84	1.31	1.86	1.67	1.77
Ramachandran favored (%)	97.44	97.45	95.79	95.33	97.22	97.22
Ramachandran allowed (%)	2.44	2.08	3.97	3.97	2.78	2.31
Ramachandran outliers (%)	0.12	0.23	0.23	0.70	0.00	0.46
Rotamer outliers (%)	1.67	0.46	0.60	8.24	2.91	3.66
Clashscore	8.71	4.15	6.03	10.77	9.80	3.78

Ligand RSCC values (A B)			0.92 0.96	0.94 0.95	0.89 0.93	0.89 0.93
B-factors						
average	32.92	51.13	38.87	74.17	39.89	48.44
macromolecules	31.51	50.49	38.74	73.97	39.86	48.00
ligands	44.20	77.90	41.30	93.89	46.35	54.72
solvent	42.67	51.44	41.55	42.14	38.24	51.37

SI Table 4. Data collection and refinement statistics

Statistics for the highest-resolution shell are shown in parentheses.

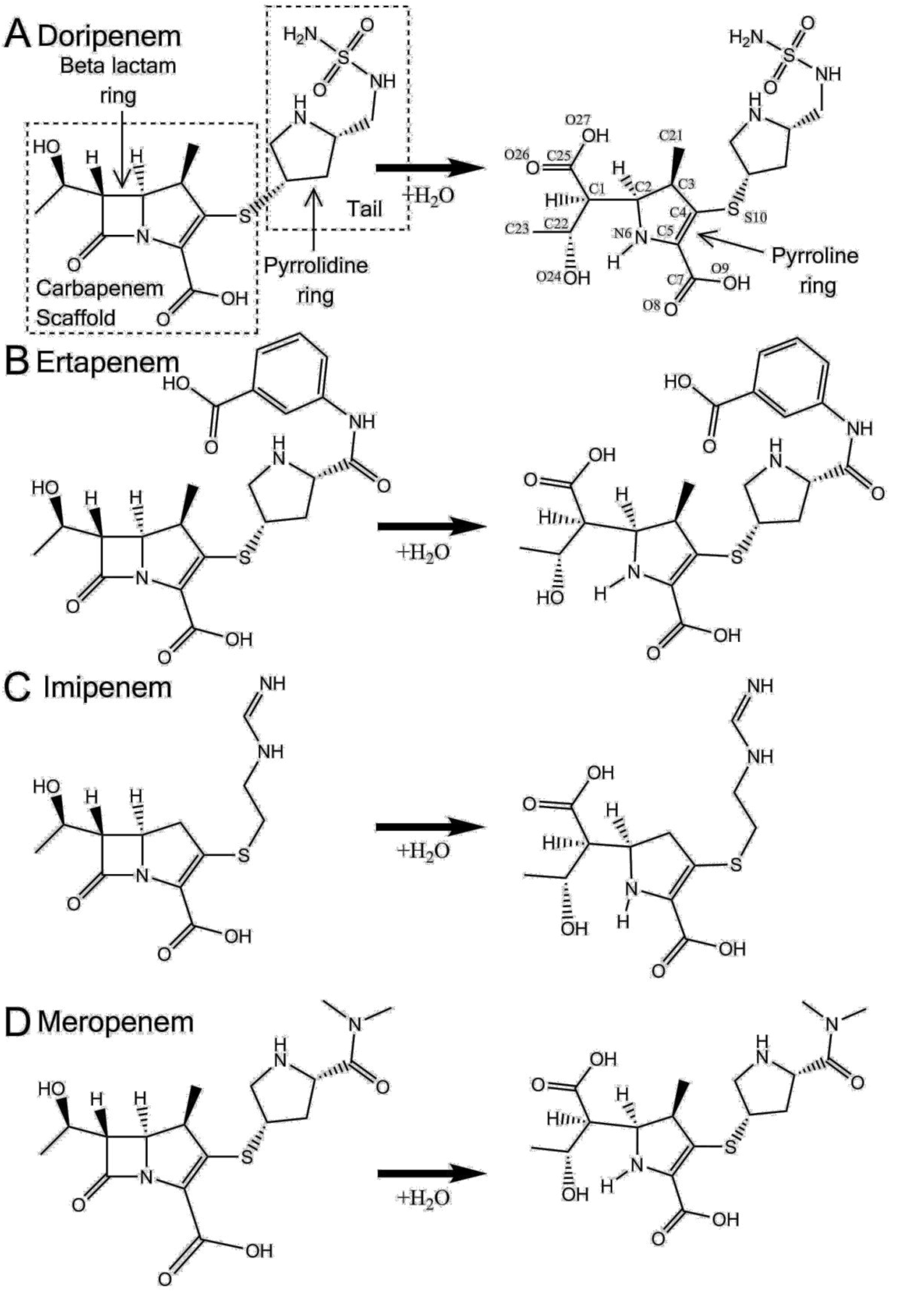

SI FIG 1. Structures of the tested carbapenems. Both hydrolyzed (right) and unhydrolyzed (left) forms of the four carbapenems are depicted, with key moieties annotated.

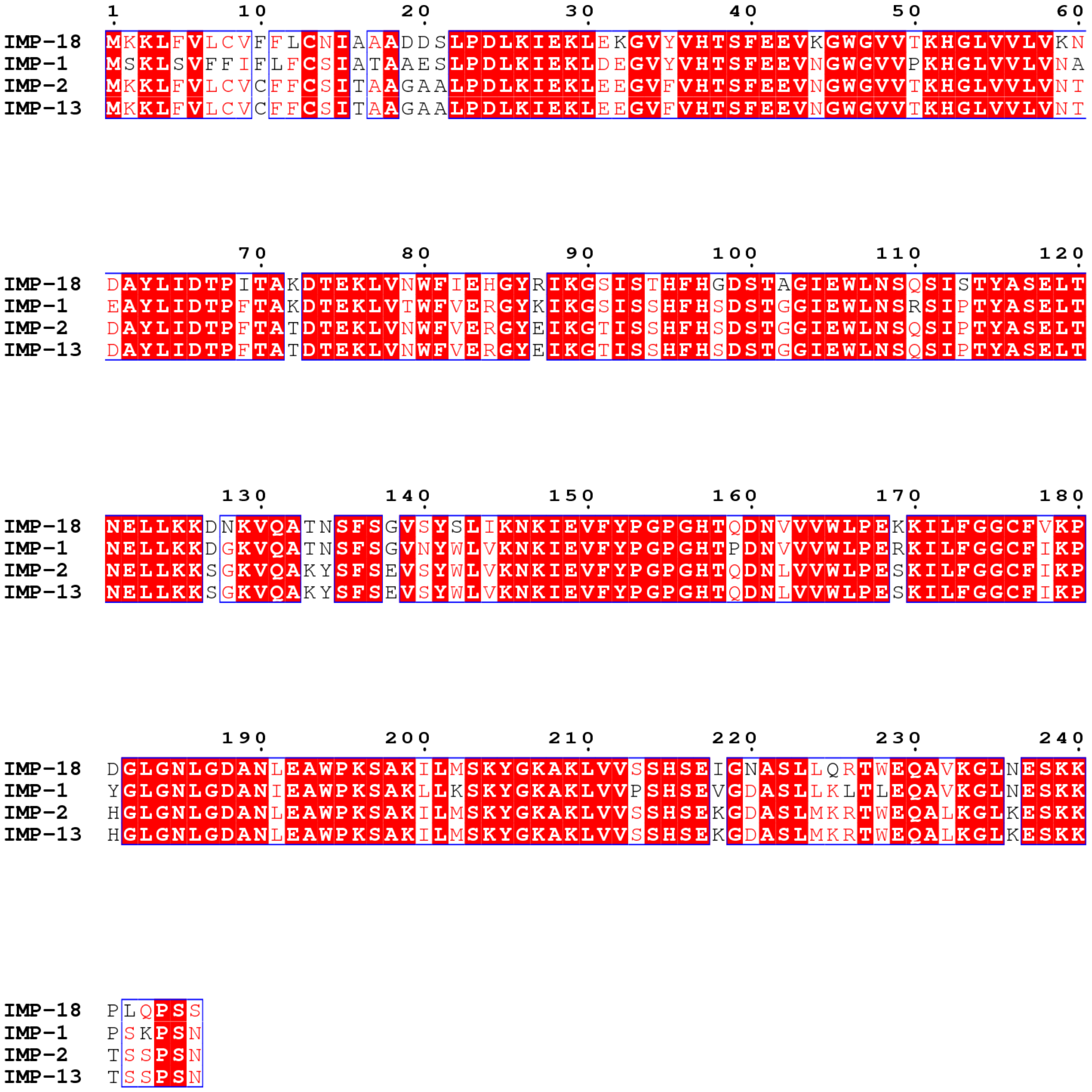

SI FIG 2. Sequence alignment of IMP-1, IMP-2, IMP-13 and IMP-18. Conserved residues are highlighted in red and partially conserved residues are shown in red text.

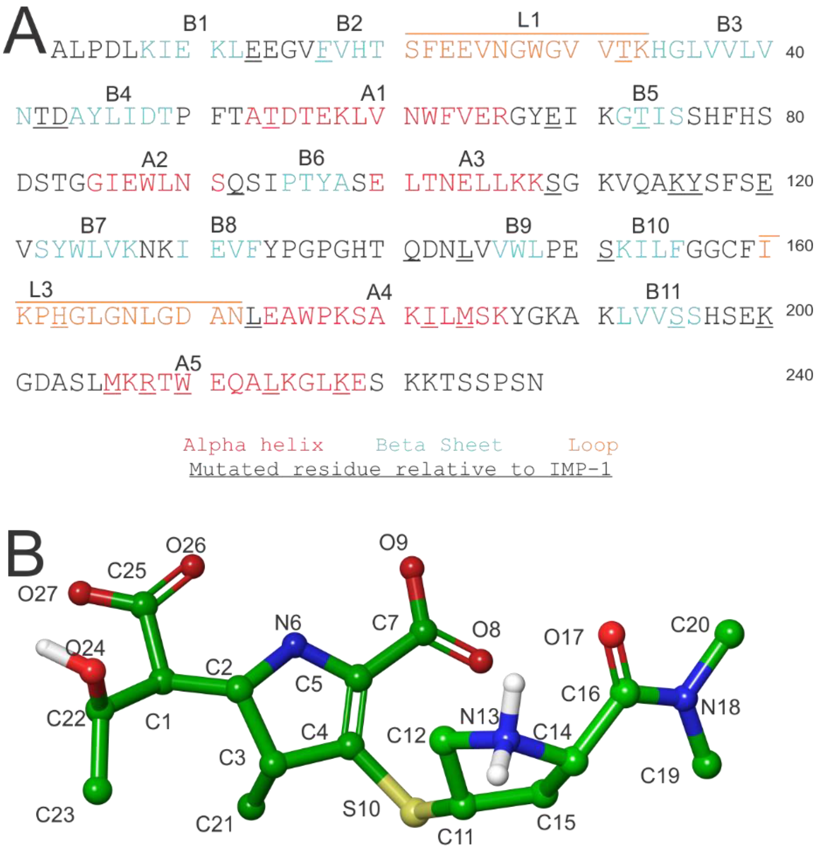

SI FIG 3. Labelled protein sequence and hydrolyzed meropenem. A) Protein sequence with secondary structure elements highlighted. L2 as in the previously published structural alignment (4) consists only of residue Lys111 and so is not marked. Residues 111 to 120 form a turn. B) Hydrolyzed meropenem structure, with numbered atoms

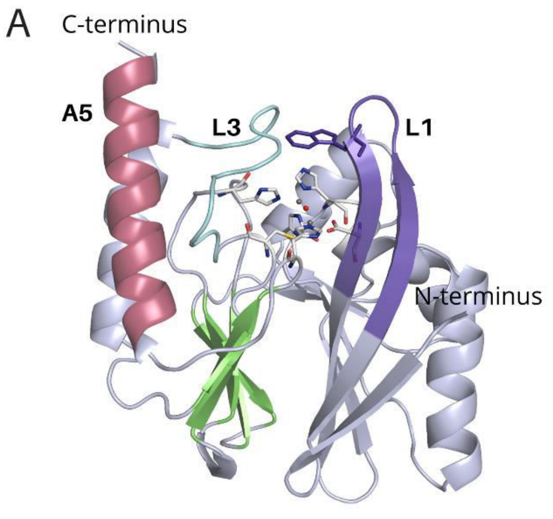

SI FIG 4. Annotated crystal structure (apo$_{\text{closed}}$). Key structural elements referred to in the text are marked.

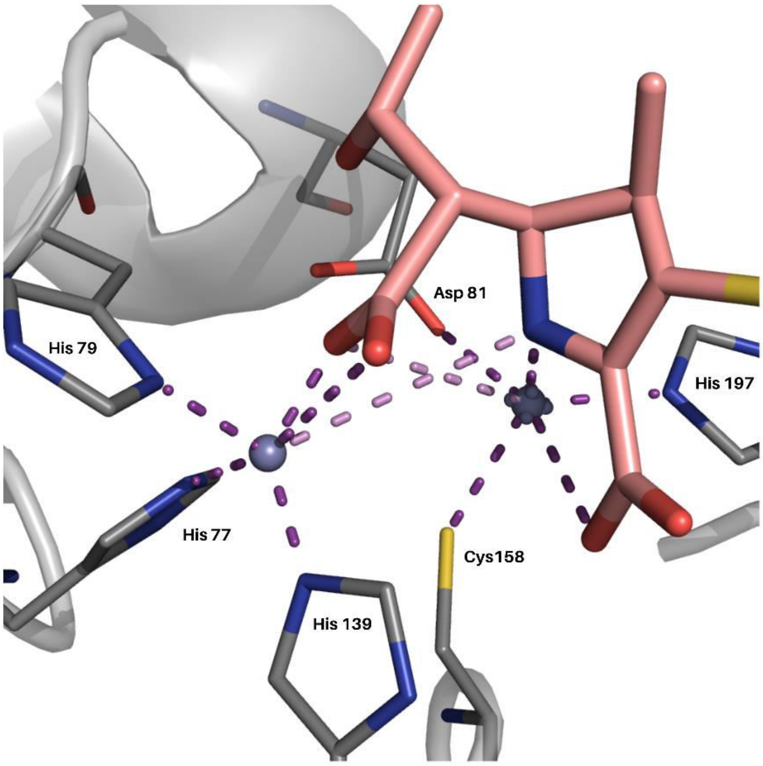

SI FIG 5. Conserved co-ordination of Zn1 and Zn2 in the carbapenem-bound state.

Meropenem is shown here as an example. Longer-distance, potentially transient, interactions are shown in light purple, tighter interactions in violet.

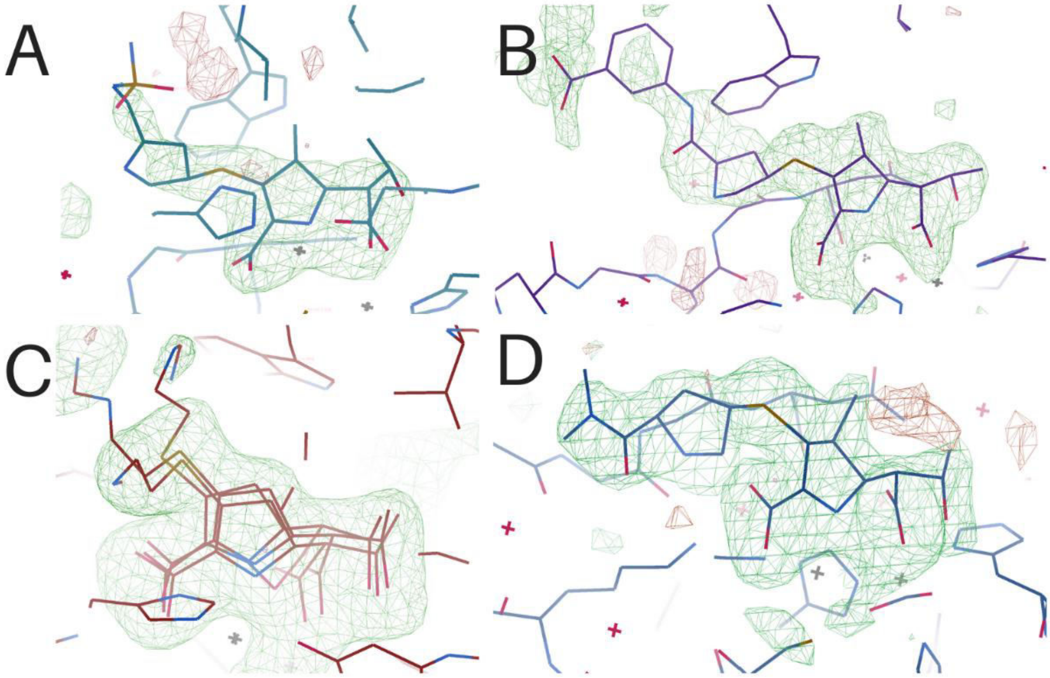

SI FIG 6. mFo-DFc omit maps. Omit maps of (A) doripenem, (B) ertapenem, (C) imipenem and (D) meropenem at 3σ.

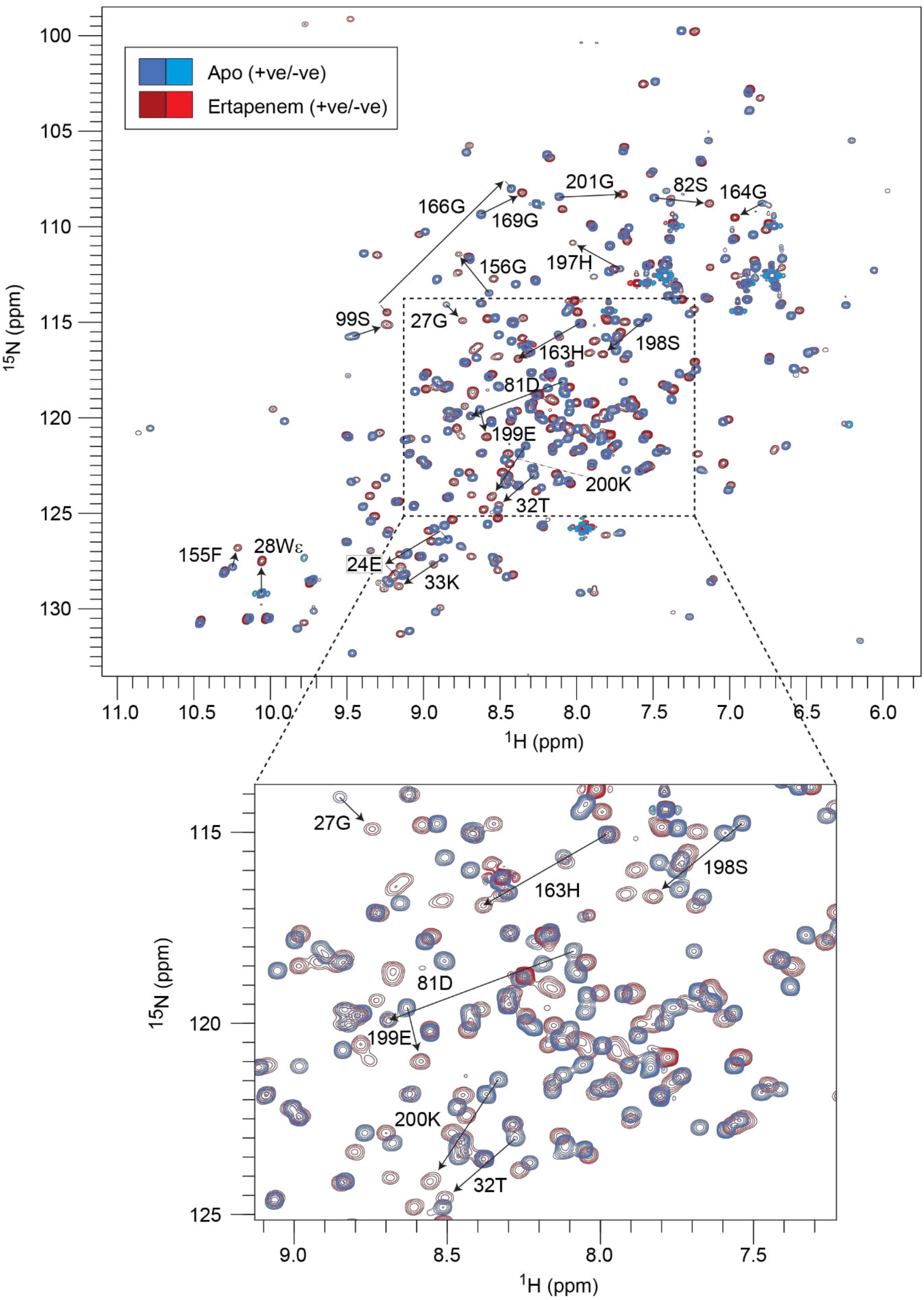
Apo (+ve/-ve)
Ertapenem (+ve/-ve)
166G
169G
201G
82S
164G
156G
197H
99S
27G
163H
198S
81D
199E
200K
32T
155F
28Wε
24E
33K
15N (ppm)
1H (ppm)

102

103

SI FIG 7. Overlay of ^{1}H,^{15}N HSQC spectra for apo and ertapenem-bound IMP-13.

(previous page) Overlay of ^{1}H,^{15}N HSQC spectra for apo-form IMP-13 (800 MHz ^{1}H frequency) and ertapenem-bound IMP-13 (600 MHz ^{1}H frequency), recorded at 25 °C. The apo spectrum is shown in blue and the ertapenem spectrum in red (positive and negative contours are shown in dark and light colours respectively). Significant chemical shift changes, marked in bright red ($\Delta\delta > 0.3$) on the structure in Figure 4B, are indicated with arrows and the assignments given.

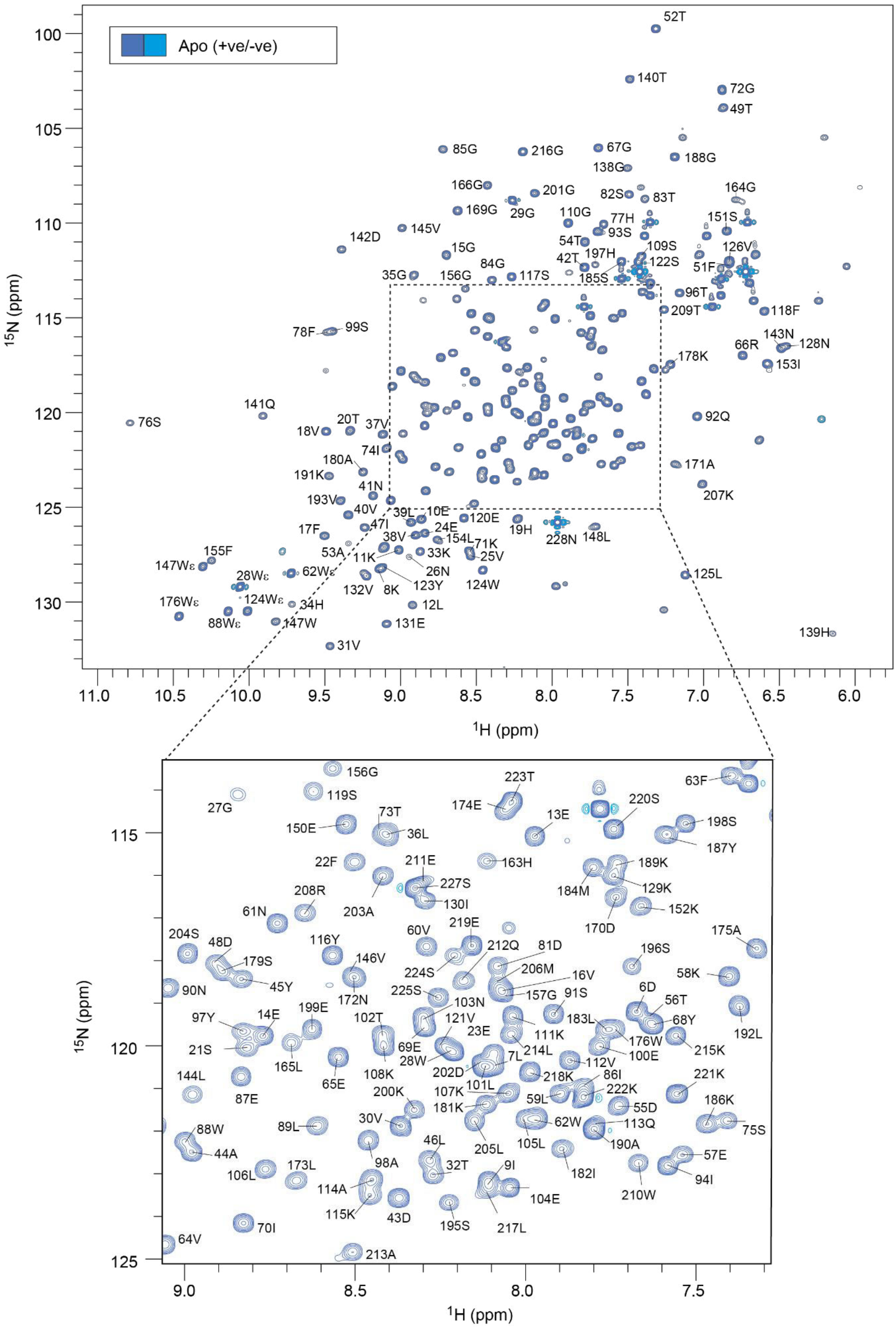

Apo (+ve/-ve)
^{15}N (ppm)
^{1}H (ppm)
100
105
110
115
120
125
130
11.0
10.5
10.0
9.5
9.0
8.5
8.0
7.5
7.0
6.5
6.0
52T
140T
72G
49T
85G
216G
67G
188G
138G
166G
201G
82S
83T
164G
169G
29G
110G
77H
151S
145V
93S
142D
54T
109S
126V
15G
197H
42T
122S
51F
84G
35G
156G
117S
185S
96T
209T
118F
78F
99S
143N
66R
128N
178K
153I
141Q
76S
20T
37V
18V
92Q
74I
180A
171A
191K
41N
207K
193V
40V
39L
10E
120E
47I
24E
19H
17F
38V
154L
228N
148L
155F
53A
71K
33K
25V
147Wε
11K
26N
62Wε
28Wε
123Y
124W
125L
132V
8K
124Wε
34H
12L
176Wε
88Wε
147W
131E
139H
31V
115
120
125
9.0
8.5
8.0
7.5
156G
223T
63F
119S
27G
174E
220S
73T
13E
150E
198S
36L
187Y
22F
211E
163H
189K
227S
184M
129K
208R
130I
203A
61N
152K
219E
170D
60V
175A
204S
48D
116Y
212Q
81D
196S
179S
146V
224S
206M
58K
16V
45Y
90N
225S
157G
91S
172N
6D
103N
56T
199E
97Y
14E
102T
121V
183L
68Y
23E
111K
176W
192L
21S
69E
214L
215K
165L
28W
7L
100E
108K
202D
218K
112V
221K
144L
86I
65E
87E
200K
101L
107K
59L
222K
186K
181K
55D
30V
62W
113Q
75S
89L
88W
105L
205L
190A
46L
44A
98A
57E
32T
9I
182I
106L
173L
94I
114A
104E
210W
115K
43D
195S
217L
70I
64V
213A

111

SI FIG 8. ^{1}H,^{15}N HSQC spectrum for apo IMP-13 showing assigned residues.

(previous page) Apo IMP-13 ^{1}H,^{15}N assignments at 25 °C, 800 MHz (^{1}H frequency) assigned using standard backbone assignment experiments (see Methods). Positive and negative contours are shown in dark and light colours respectively.

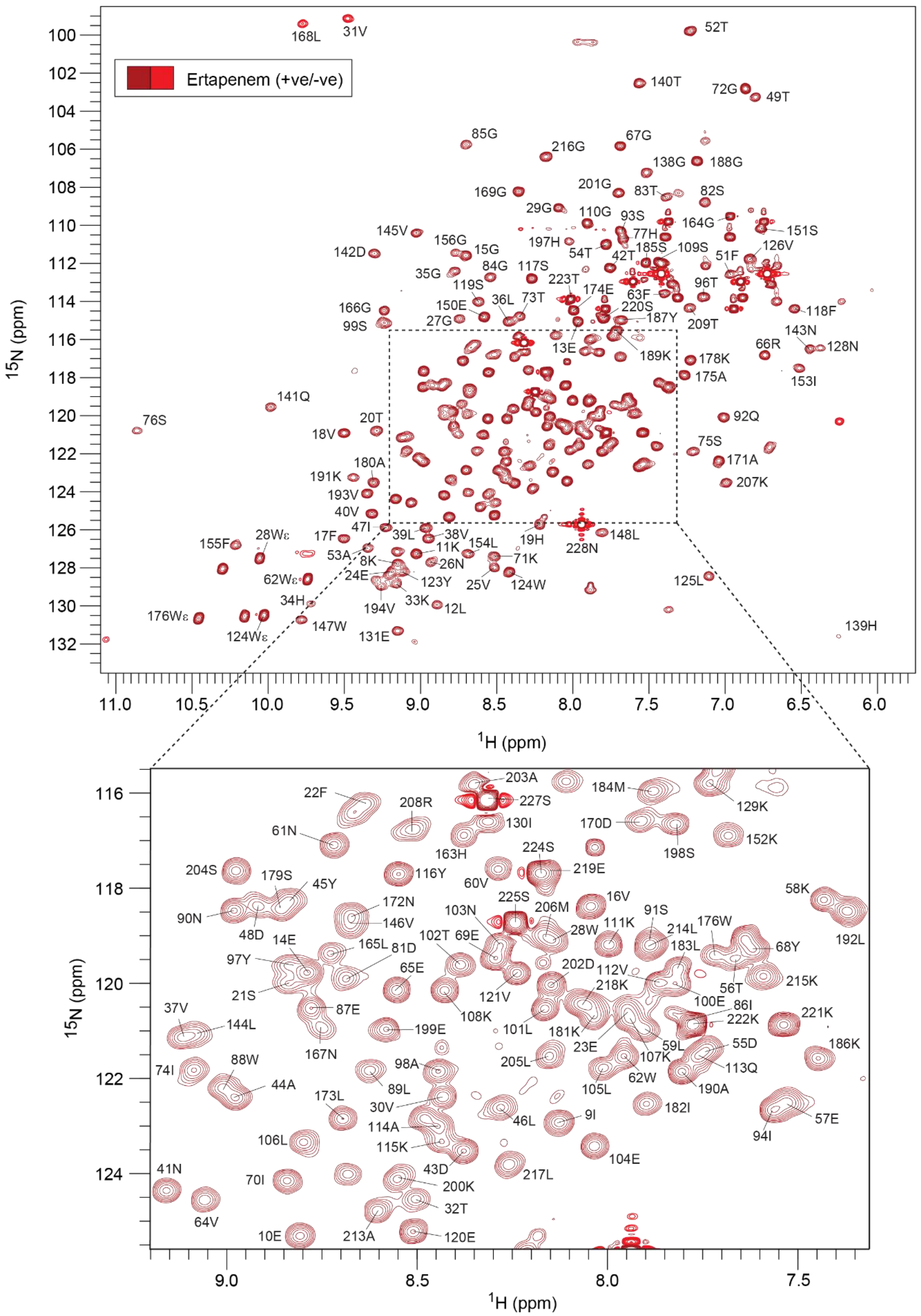
Ertapenem (+ve/-ve)
15N (ppm)
1H (ppm)
100
102
104
106
108
110
112
114
116
118
120
122
124
126
128
130
132
11.0
10.5
10.0
9.5
9.0
8.5
8.0
7.5
7.0
6.5
6.0
168L
31V
52T
140T
72G
49T
85G
216G
67G
138G
188G
201G
83T
82S
169G
29G
110G
93S
164G
151S
145V
77H
109S
126V
156G
197H
54T
42T
185S
51F
142D
15G
84G
117S
35G
223T
96T
119S
174E
63F
36L
73T
150E
220S
118F
166G
27G
187Y
209T
99S
143N
128N
66R
13E
189K
178K
175A
153I
141Q
120
76S
20T
92Q
18V
75S
171A
180A
207K
191K
193V
40V
47I
39L
38V
148L
155F
28Wε
17F
11K
154L
19H
228N
53A
71K
8K
26N
62Wε
24E
123Y
25V
124W
125L
34H
33K
194V
12L
176Wε
124Wε
147W
139H
131E
22F
203A
184M
208R
227S
129K
61N
130I
170D
152K
163H
224S
198S
204S
179S
45Y
116Y
219E
60V
58K
16V
90N
172N
225S
103N
206M
91S
146V
176W
111K
214L
192L
48D
14E
165L
81D
102T
69E
28W
183L
68Y
97Y
65E
202D
112V
218K
56T
215K
21S
121V
100E
86I
37V
87E
108K
101L
181K
222K
221K
144L
199E
23E
59L
55D
186K
167N
205L
107K
113Q
74I
88W
98A
62W
105L
190A
44A
89L
173L
30V
46L
9I
182I
57E
94I
114A
106L
115K
104E
43D
41N
217L
70I
200K
64V
32T
10E
213A
120E

SI FIG 9. ^{1}H,^{15}N HSQC spectrum for ertapenem-bound IMP-13 showing assigned residues.

(previous page) Ertapenem-bound IMP-13 ^{1}H,^{15}N assignments at 25 °C, 600 MHz (^{1}H frequency) assigned using standard backbone assignment experiments (see Methods). Positive and negative contours are shown in dark and light colours respectively.

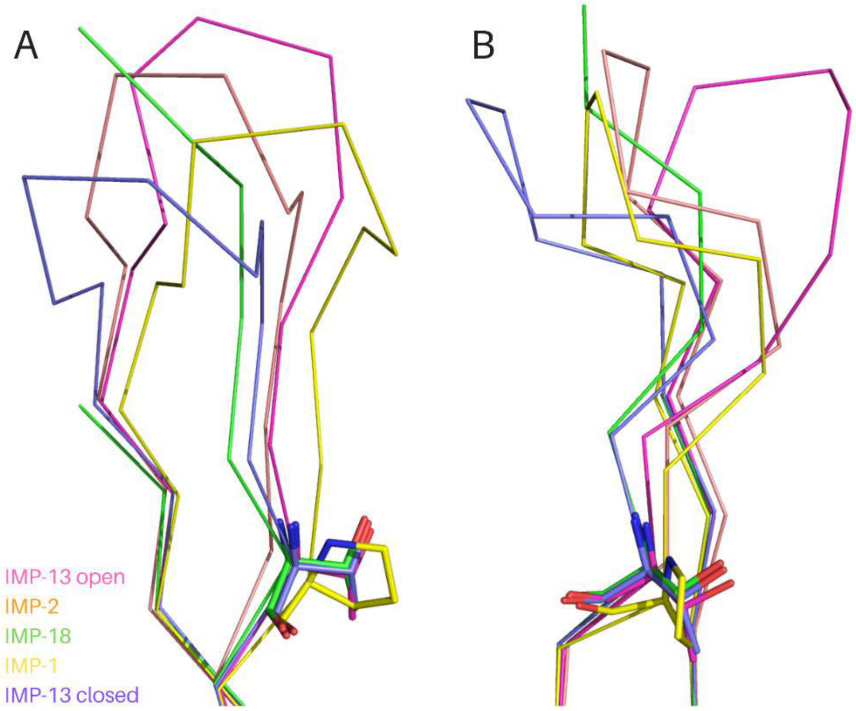

SI FIG 10. Loop 1 of different IMP variants. IMP-13 closed (purple) and open (magenta) forms, in comparison with IMP-18 (5B3R (5), green, not fully modelled), IMP-1 (5Y5B (6), yellow) and IMP-2 (4UBQ (7), peach), with residue 32 shown in sticks (proline in IMP-1 shown in yellow). (A) Front view, (B) Side view

Supplemental references

1. **Laraki N, Franceschini N, Rossolini GM, Santucci P, Meunier C, Pauw Ed, Amicosante G, Frère JM, Galleni M.** 1999. Biochemical Characterization of the Pseudomonas aeruginosa 101/1477 Metallo-β-Lactamase IMP-1 Produced by Escherichia coli. Antimicrobial Agents and Chemotherapy **43:**902-906.
2. **Santella G, Docquier J-D, Gutkind G, Rossolini GM, Radice M.** 2011. Purification and Biochemical Characterization of IMP-13 Metallo-β-Lactamase. Antimicrobial Agents and Chemotherapy **55:**399-401.
3. **Yong D, Toleman MA, Giske CG, Cho HS, Sundman K, Lee K, Walsh TR.** 2009. Characterization of a New Metallo-β-Lactamase Gene, blaNDM-1, and a Novel Erythromycin Esterase Gene Carried on a Unique Genetic Structure in *Klebsiella pneumoniae* Sequence Type 14 from India. Antimicrobial Agents and Chemotherapy **53:**5046–5054.
4. **Garau G, García-Saez I, Bebrone C, Anne C, Mercuri P, Galleni M, Frere J-M, Dideberg O.** 2004. Update of the Standard Numbering Scheme for Class B β-Lactamases. Antimicrobial Agents and Chemotherapy **48:**2347–2349.
5. **Furuyama T, Nonomura H, Ishii Y, Hanson ND, Shimizu-Ibuka A.** 2016. Structural and Mutagenic Analysis of Metallo-β-Lactamase IMP-18. Antimicrobial Agents and Chemotherapy **60:**5521-5526.
6. **Wachino J-i, Kanechi R, Nishino E, Mochizuki M, Jin W, Kimura K, Kurosaki H, Arakawa Y.** 2019. 4-Amino-sulphanylbenzoic acid as a potent subclass B3 metallo-beta-lactamase-

specific inhibitor applicable for distinguishing metallo-beta-lactamase subclasses. Antimicrob Agents Chemother **63:**e01197-01119.

7. **Yamaguchi Y, Matsueda S, Matsunaga K, Takashio N, Toma-Fukai S, Yamagata Y, Shibata N, Wachino J-i, Shibayama K, Arakawa Y, Kurosaki H.** 2015. Crystal Structure of IMP-2 Metallo-β-lactamase from Acinetobacter spp. Biol Pharm Bull **38:**96-101.
